# A practical approach for adoption of a hub and spoke model for cell and gene therapies in low- and middle-income countries: framework and case studies

**DOI:** 10.1038/s41434-023-00425-x

**Published:** 2023-10-30

**Authors:** Shadi Saleh, Omar Dabbous, Sean D. Sullivan, Dipen Ankleshwaria, Daiane Trombini, Mondher Toumi, Mahmoud Diaa, Anish Patel, Burcu Kazazoglu Taylor, Sean Tunis

**Affiliations:** 1 American University of Beirut, Beirut, Lebanon; 2 Novartis Gene Therapies, Inc., Bannockburn, IL USA; 3 CHOICE Institute, School of Pharmacy, University of Washington, Seattle, WA USA; 4 Novartis, Dubai, United Arab Emirates; 5 Novartis, Miami, FL USA; 6 Aix Marseille University, Marseille, France; 7 Novartis, Singapore, Singapore; 8 Novartis Gene Therapies, Washington, DC USA; 9 Rubix Health, Baltimore, MD USA

**Keywords:** Gene therapy, Gene regulation

## Abstract

In the rapidly evolving landscape of biotechnologies, cell and gene therapies are being developed and adopted at an unprecedented pace. However, their access and adoption remain limited, particularly in low- and middle-income countries (LMICs). This study aims to address this critical gap by exploring the potential of applying a hub and spoke model for cell and gene therapy delivery in LMICs. We establish the identity and roles of relevant stakeholders, propose a hub and spoke model for cell and gene therapy delivery, and simulate its application in Brazil and the Middle East and North Africa. The development and simulation of this model were informed by a comprehensive review of academic articles, grey literature, relevant websites, and publicly available data sets. The proposed hub and spoke model is expected to expand availability of and access to cell and gene therapy in LMICs and presents a comprehensive framework for the roles of core stakeholders, laying the groundwork for more equitable access to these lifesaving therapies. More research is needed to explore the practical adoption and implications of this model.

## Introduction

Cell and gene therapies (CGTs) are in the vanguard of modern medicine. There are more than 3500 CGTs currently in development targeting a wide array of diseases, including cancer, amyotrophic lateral sclerosis, sickle cell disease, and acquired immune deficiency syndrome, as well as many rare genetic diseases [1]. In the context of this study, we define gene therapy as the therapeutic delivery of nucleic acid into a patient’s cells to treat disease, and cell therapy as the administration of living cells into a patient’s body to treat or cure disease. These broad definitions encompass treatments for a range of diseases that are responsive to CGTs.

By 2030, it is projected that up to 60 new CGTs could be launched that would affect more than 350,000 patients, making such therapies one of the most impactful areas of research and investment in medicine [2–4]. The adoption of such therapies is accompanied by a multitude of challenges, and the stakeholders, processes, and outputs involved in CGTs are quite different than traditional pharmaceuticals, necessitating updated approaches for planning, manufacturing, and delivery [5]. A key challenge common to all highly specialized care, including CGTs, is to deliver quality health services to a specific, often small, number of geographically dispersed patients. This is particularly relevant with CGTs, given the associated infrastructural and therapeutic costs from the demand and supply sides. The conventional approach of concentrating clinical, logistical, and infrastructural expertise and capacities in a few large centers of care is not conducive for optimized delivery of CGTs. In low- and middle-income countries (LMICs) in particular, multiple barriers exist to the access to and delivery of CGTs, including inequitable healthcare access, lack of resources, funding shortages, the prohibitive cost of CGTs, and complex regulatory systems [6, 7]. Furthermore, CGT manufacturing, utilization, and access is concentrated in high-income countries. As of 2021, only three of the 20 approved gene therapies worldwide were approved in LMICs [1, 8]. This inequitable landscape of CGTs is worrying, especially given that LMICs carry approximately 90% of the global burden of disease [9]. Concerted efforts are required to expand the delivery of CGTs in LMICs; otherwise, the divide between the disease burden and therapeutic availability will keep growing.

Although it is true that many of the challenges faced by the administration of CGTs are common to other advanced treatments such as bone marrow transplants, CGTs have unique aspects that set them apart. These therapies are often highly personalized, requiring the modification of the patient’s own cells or the design of a therapeutic genetic sequence specific to the patient’s condition. This personalized nature poses unique manufacturing and logistical challenges. Moreover, many CGTs hold the promise of being one-time treatments that can provide lasting benefits, which brings new considerations for cost-effectiveness analyses and reimbursement models. Importantly, CGTs hold substantial potential benefits for LMICs, particularly for diseases prevalent in these areas. For example, gene therapy for β-thalassemia, a prevalent inherited disorder in LMICs, has demonstrated promising results in restoring hemoglobin production and reducing the need for blood transfusions [10]. These unique aspects necessitate specific considerations in the planning, delivery, and post-administration management of CGTs, especially in LMICs. The need to adapt traditional approaches or consider and adopt novel approaches in delivering such therapies is great.

One such approach is the adoption of the hub and spoke healthcare model for delivering CGTs. In a hub and spoke model for healthcare delivery, a main health facility (hub), which receives the most resources and delivers the most intensive services, is complemented with less complex health facilities (spokes), which offer a more limited array of services [5, 11]. Some hub and spoke models also include a partner spoke, a smaller health facility aimed at expanding access to the health services provided by hubs and spokes, especially in rural areas [12]. This model is scalable, efficient, and most importantly, adaptable based on needs and context [13]. The model has been implemented in cancer care to distribute services and improve patient outcomes, in pain management to promote telehealth programs and reach underserved areas, and in acute stroke care to enable remote consultation and timely treatment [12, 14–17]. In the context of CGTs, traditional delivery models have focused on manufacturing of therapeutic products without full consideration of delivery and access [18, 19]. Recently, calls for delivery of gene therapy, specifically adeno-associated virus–based gene therapy, through hub and spoke models have been observed [20–22]. We propose the application of a hub and spoke model for CGT delivery in LMICs, accompanied with a developed framework for core CGT stakeholders. This proposed hub and spoke model is intended to address a wide spectrum of these diseases, which includes inherited conditions, such as certain metabolic disorders, hemophilia, and muscular dystrophy, in which mutations in a single gene can be targeted. It also includes diseases such as cancer, in which gene therapy can be used to modify immune cells to recognize and attack cancer cells, and viral infections, in which gene therapy can potentially be used to target and eliminate the viral genome. In the context of cell therapies, our model may address conditions including cancers in which modified T cells (like CAR-T cells) can be used and autoimmune diseases in which regulatory T cells can potentially restore immune tolerance. This model is then simulated in two distinct scenarios in LMICs: a within-country scenario in Brazil, and a cross-country scenario in the Middle East and North Africa (MENA).

There are a number of emerging treatments that could benefit from our proposed CGT delivery model. Advances in gene editing technologies, such as CRISPR-Cas9, are promising for addressing genetic diseases at their source. Other gene therapies, such as those targeting hemophilia, have already received approval in the Unites States [23], and clinical trials are also being conducted in Brazil. Similarly, CAR-T cell therapies are revolutionizing the treatment of certain cancers and may soon become more prevalent [24–26]. Our proposed model could serve as a vehicle to ensure these treatments reach patients in LMICs, further underscoring the relevance and potential of our study.

### CGT delivery in Brazil

According to the American Society of Gene & Cell Therapy (ASCGT), 21 CGT clinical trials, nine of which are in phase 3, are underway in Brazil, which is an LMIC as defined by the World Bank [1, 27]. The first CGT-related clinical trial in Brazil was conducted in the early 2000s, after the government developed and implemented more than 10 cellular therapy research centers in recognition of the potential of CGTs [28]. Later developments in the field eventually led to the recent resolution by the National Health Surveillance Agency (Agência Nacional de Vigilância Sanitária, ANVISA) aimed at regulating the research, development, clinical application, and registration of advanced therapeutic products in Brazil. More recently, ANVISA, in collaboration with the United Nations Development Program, called for the development of the National Network of Specialists in Advanced Therapies (Rede Nacional de Especialistas em Terapias Avançadas, RENETA), a network of experts that support the evaluation of clinical trials, product registration, and monitoring processes of advanced therapeutic products. RENETA also assists in building human capacity at ANVISA to ensure proper regulation over advanced therapeutic products [29]. Thus, Brazil represents a country that is actively engaging with the CGT space. The government’s proactive approach to the development of cellular therapy research centers and its recent regulatory resolutions demonstrates a strong commitment to facilitating the adoption of CGTs.

### CGT delivery in the MENA region

CGT delivery is still in its infancy in the MENA region. The first record of gene therapy administration in this region dates to 2018, where the first dose of nusinersen, a gene therapy product for patients with spinal muscular atrophy, was administered at the East Jeddah Hospital in Saudi Arabia [30]; the drug is now approved in Kuwait, Qatar, and the United Arab Emirates (UAE) [31]. In 2019, voretigene neparvovec, a gene therapy treatment for inherited retinal blindness, was approved by the Ministry of Health and Prevention and the Saudi Food and Drug Authority in the UAE and Saudi Arabia, respectively [32, 33]. Another gene therapy product, onasemnogene abeparvovec, was used to treat children with spinal muscular atrophy in Egypt, Saudi Arabia, Kuwait, Qatar, and the UAE [34–39]. Outside of North America, the Hamad General Hospital in Qatar was the first to administer this drug. For cell therapy products, tisagenlecleucel, a chimeric antigen receptor T-cell therapy product, was approved by the Saudi Food and Drug Authority in 2022 [40]. This reflects the variability in resources and capacities across the MENA region. Indeed, according to the World Bank, the MENA region includes high-income (Bahrain, Kuwait, Oman, Qatar, Saudi Arabia, UAE) and low- and middle-income countries (Lebanon, Djibouti, Egypt, Iran, Iraq, Morocco, Tunisia, Jordan, Libya, Morocco, Syria, Tunisia, West Bank and Gaza, Yemen) [27].

## Materials and Methods

### Stakeholder framework and mapping

The core stakeholder framework and mapping for CGT delivery was informed by the existing literature in addition to the websites and online documentation of relevant pharmaceutical, governmental, academic, and information technology sectors. A major source of information, BioPhorum, a leading business-to-business membership organization with more than 6000 subject matter experts, has developed more than 30 documents on CGT. These documents include a detailed analysis and description of CGT-related challenges [41], considerations [42], and more importantly, process maps [43, 44] and actors [45]. Another example is the joint partnership between the European Association for Hemophilia and Allied Disorders and the European Hemophilia Consortium to develop a framework for a hub and spoke model for delivery of hemophilia CGT [46].

In Brazil, facilities for cell processing were extracted from a data set on cell processing centers [47] and the National Cell Therapy Network (Rede Nacional de Terapia Celular, RNTC) website [48]. Facilities involved in CGT clinical trials were extracted from the literature, clinicaltrials.gov [2], the RENETA website [29], and the ASCGT website [1]. Information on potential CGT manufacturers in Brazil was extracted from CGT clinical trial sponsors, which may or may not be the CGT manufacturer, in addition to the literature and governmental or pharmaceutical websites in Brazil [49–51]. To decide whether the health facilities identified were academic centers, the corresponding website and SUS (Sistema Único de Saúde) information page of the site were reviewed. Accreditation of health facilities was reviewed using the Foundation for the Accreditation of Cellular Therapy and the Association for the Advancement of Blood & Biotherapies/Associação Brasileira de Hematologia, Hemoterapia e Terapia Celular accreditation websites [52, 53]. Health facilities able to perform hematopoietic stem cell transplantation were extracted from DATASUS data set [54].

In the MENA region, cell processing centers were extracted from data sets, websites, and publications on cord blood banks and facilities in the Middle East [55–59]. It is important to note that cord blood banks may or may not have the capacity to process cells for cell therapies. Facilities involved in CGT clinical trials were extracted from the literature as well as the clinicaltrials.gov website [2] and the ASCGT website [1]. Information on potential CGT manufacturers in the MENA region was extracted from CGT literature and governmental/pharmaceutical websites in the region. Only two pharmaceutical companies in Saudi Arabia were identified, Saudi Vax and Sudair Pharma [60, 61]. The Abu Dhabi Stem Cell Center in the UAE is also advertised to locally engineer chimeric antigen receptor T-cells [62]. The sponsors identified in CGT clinical trials in the MENA region are multinational companies with offices in the Middle East, but information on whether such companies manufacture CGT products in the region was not available.

### Shortlisting hubs, spokes, and partner spokes for simulation

Given that CGT delivery is still in its infancy in Brazil, CGT clinical trials are a rich source of information for simulating the CGT hub and spoke model. Sites that can perform and lead clinical trials have the capacity to administer CGTs. Thus, the health facilities involved in CGT clinical trials were shortlisted as hubs. One of the main criteria suggested for a CGT spoke is having the clinical capacity to administer at least one type of CGT therapy and host processing facilities. Bone marrow transplantation requires collection, processing, cryopreservation, and reinfusion, which are processes similar to CGT delivery. Thus, to identify potential CGT spokes, a list of hospitals that provide bone marrow transplantation services in Brazil were extracted. Those that provide both allogenic and autogenic bone marrow transplantation were shortlisted, and those that were listed as hubs were filtered out. Potential CGT partner spokes were extracted from the DATASUS data set, particularly health facilities that have hematological and/or hemotherapy care units. This would satisfy two main criteria of CGT partner spokes: the capacity to perform apheresis and the presence of qualified care professionals. Importantly, many hematological and/or hemotherapy care units are hosted within basic health units, which are highly embedded within communities in Brazil.

In the MENA region, consolidated data sets on types of facilities and associated services were absent, and fragmented data from international and governmental websites were core sources. In addition to health facilities involved in CGT clinical trials, health facilities in the MENA region that already provide CGT services were extracted through literature search and news, as well as governmental and nongovernmental websites [62–65]. These were also included in the list of potential hubs. For potential spokes, a partial list of bone marrow transplantation centers in the MENA region was retrieved from the European Society for Blood and Marrow Transplantation and the Eastern Mediterranean Blood and Marrow Transplantation membership documentation [66, 67]. Other centers were extracted through literature search, websites for health facilities, and governmental and social media sites [68–75]. The final list of 37 potential spokes was produced after filtering out hospitals that were already shortlisted as hubs. Because of an absence of data sets on services provided by health facilities in the MENA region, potential CGT partner spokes were considered to be the rest of the hospitals that were not shortlisted as hubs or spokes. The rationale is that these hospitals, many of which are municipal, are well embedded into communities in the MENA region and would have qualified personnel for potential apheresis. Importantly, these facilities would also have the capacity to screen patients and refer them to spokes.

For both Brazil and the MENA region, shortlisted hub, spoke, and partner spoke criteria and respective health facilities were coded when producing the matrices to allow for a more streamlined assessment.

### Geographic mapping

All GIS mapping was done using QGIS, a free and open-source cross-platform desktop geographic information system application that supports viewing, editing, printing, and analysis of geospatial data [76]. In the simulation models, a distance to point function was used in QGIS to measure the closest distance between the clinical trial sites and manufacturer. Sites that were within 20 kilometers of a manufacturer were considered physically close.

## Results

### Developing a framework for core CGT stakeholders for CGT delivery

**Fig. 1:**
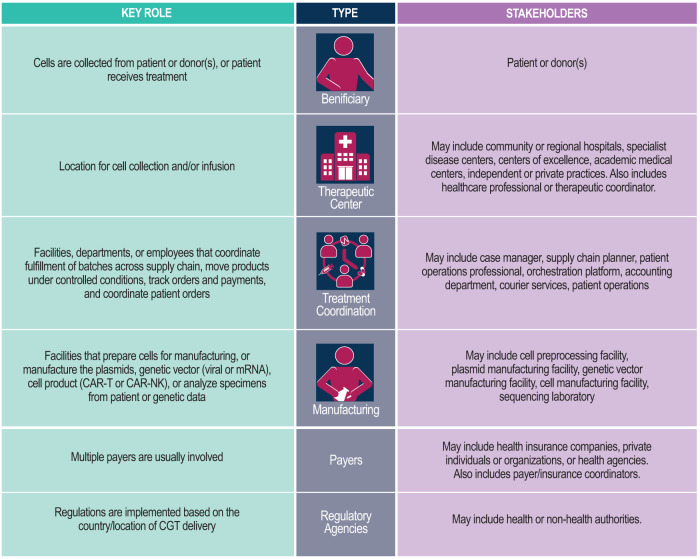
Proposed framework for CGT delivery stakeholders and their roles. Core stakeholders for CGT delivery are classified by their role, type, and identity. CGT cell and gene therapy.

The evolution of the current linear and centralized delivery model of CGT into a nonlinear hub and spoke model requires a detailed understanding and mapping of the current and potential roles of relevant stakeholders. The stakeholders involved in the different categories of CGT (i.e., cell therapies, gene modified cell therapies, gene therapies, and tissue-engineered products [10]) are variable. It is therefore important to draw on all of these to formulate a common framework of stakeholders with their core roles. The proposed framework includes four direct and two indirect CGT delivery stakeholder types (Fig. 1, Supplementary Material).

Core CGT stakeholders include beneficiaries (patients or donors), therapeutic centers, treatment coordination actors, manufacturing stakeholders, payers, and regulatory agencies. A therapeutic center collects specimens and performs pretesting on patients to inform eligibility, initiates the supply chain, prepares the patient for therapy, and administers the therapy. Treatment coordination involves planning, patient operations, accounting, an orchestration platform, and courier services to ensure proper scheduling, quality, and risk assessment of the supply process. Manufacturing analyzes specimens, prepares cells, and develops and tests therapeutic products using sequencing labs, cell preprocessing facilities, and vector manufacturing facilities. Coordination between one or more payers initiates the CGT therapeutic process, and regulatory agencies authorize and monitor the CGT pipeline. Process flow mapping for the various types of CGTs is presented in Supplementary Fig. 1 (stock genetic vector), Supplementary Fig. 2 (personalized gene therapy), Supplementary Fig. 3 (allogenic cell therapy), and Supplementary Fig. 4 (autologous cell therapy).

### The hub and spoke model for CGT delivery

The proposed hub and spoke model for CGT delivery comprises three main interconnected components: hub, spoke, and partner spoke. Briefly, the CGT hub is a leading academic medical center that is experienced in both comprehensive care and delivering CGT. A spoke is a healthcare center that has minimal CGT experience but will serve as the home center for patients. A partner spoke is a supporting facility that is not necessarily a health center but facilitates the function of spokes within the system (Supplementary Material).

**Fig. 2:**
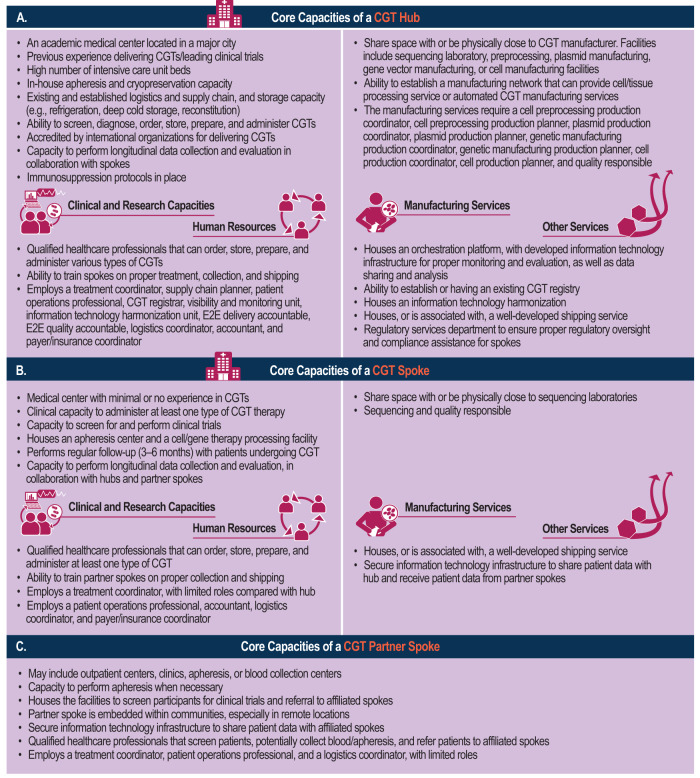
Core capacities of a CGT hub, spoke, and partner spoke. The CGT hub (**A**) is an advanced medical center specializing in CGT research and practice, with the therapeutic center having the capacity for screening, diagnosing, and treating all categories of CGT. The CGT spoke (**B**) is responsible for administering at least one type of CGT, with apheresis centers housed in spokes. The CGT partner spoke (**C**) operates in small communities without full accreditation, serving as screening, referral, and collection centers to optimize cell transit time and simplify logistics. CGT cell and gene therapy, E2E end-to-end, IT informational technology.

The CGT hub is an advanced medical center specializing in CGT research and practice, with the therapeutic center having the capacity for screening, diagnosing, and treating all categories of CGT (Fig. 2A). The hub also possesses cryopreservation and apheresis facilities, as well as co-located manufacturing facilities, and works in collaboration with biotech and pharmaceutical companies. It should have an established logistics and supply chain, with compliant manufacturing and an existing CGT registry, and offer training to HCPs. Hub and spoke personnel should collaborate to ensure optimal patient outcomes, and the orchestration platform should include a visibility and monitoring unit and an information technology harmonization unit.

The CGT spoke is responsible for administering at least one type of CGT, with apheresis centers housed in spokes. A spoke should have a cell/tissue processing facility, optimize donor starting material, and provide referral routes to hubs. The spokes also act as donation centers and should standardize treatment protocols and process flows in coordination with the hub. A treatment coordinator should be stationed at spokes, and HCPs should oversee patient information sharing (Fig. 2B).

The CGT partner spoke operates in small communities without full accreditation, serving as screening, referral, and collection centers to optimize cell transit time and simplify logistics (Fig. 2C). Outpatient centers and clinics aid in increasing referrals to spokes for CGTs and increasing access to clinical trial participants. Partner spokes should have qualified personnel, and collection centers at partner spokes may coordinate with spokes for proper shipping and initiation of the manufacturing process.

A summary of the capacities of core stakeholders required at the hubs, spokes, and partner spokes, including beneficiaries, therapeutic centers, treatment coordination, and manufacturing, is provided in Supplementary Tables 1–4.

### Simulation of the hub and spoke model in Brazil and the MENA region

The theoretical clinical utility and cost effectiveness of a hub and spoke model for delivering CGT does not guarantee its successful adoption. It is thus imperative to simulate institutional readiness for potential adoption of such models. To pressure test the hub and spoke institutional criteria developed, we simulated the adoption of a within-country hub and spoke model of CGT delivery in Brazil and a cross-country model of CGT delivery in the MENA region.

**Fig. 3:**
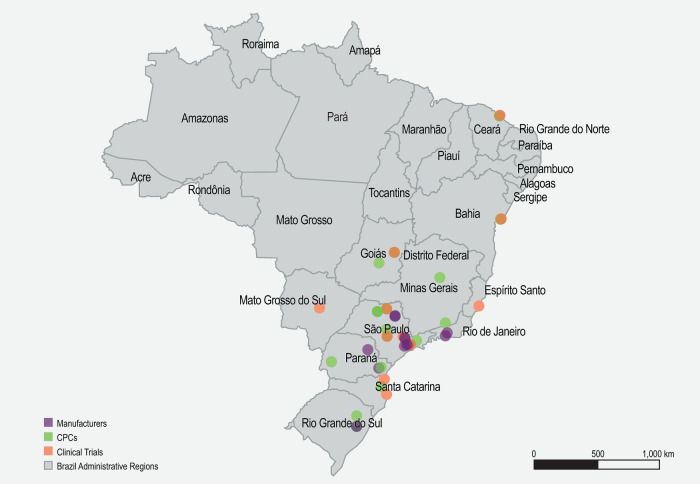
Geographical distribution of facilities involved in CGT clinical trials, cell processing centers, and potential manufacturers in Brazil. CGT Cell and gene therapy, CPC Cell processing center.

**Fig. 4:**
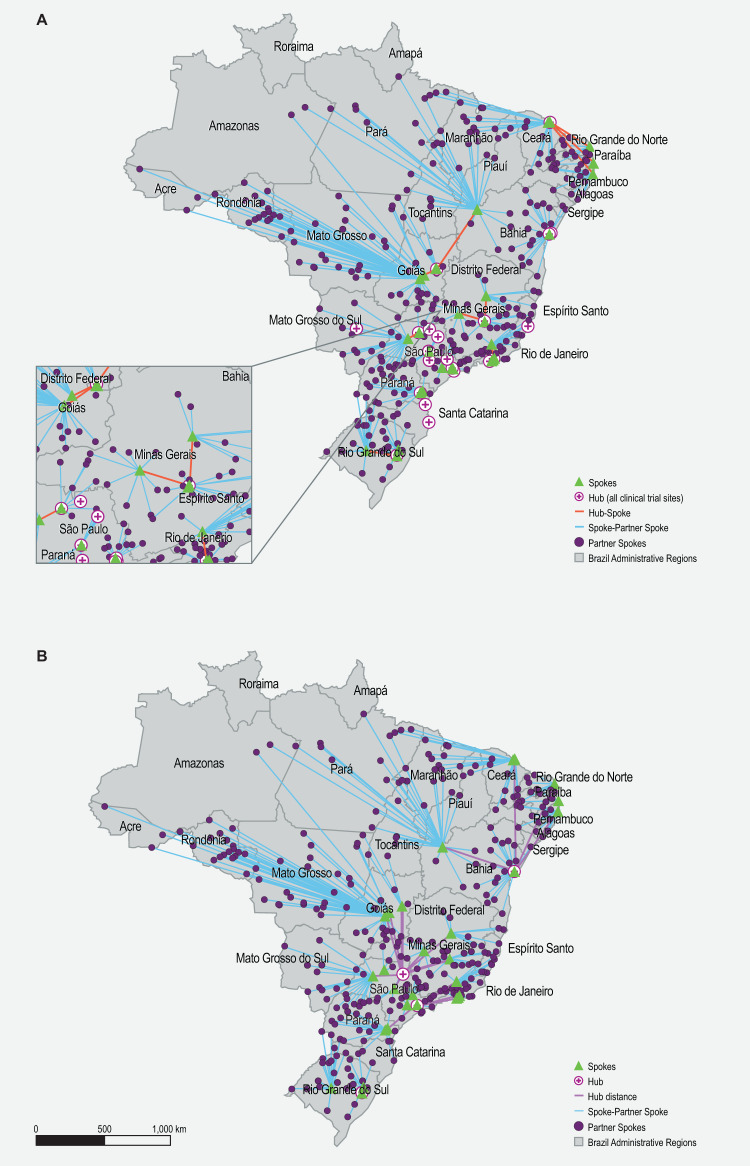
Hub and spoke simulation for Brazil. All clinical trial sites are presented as hubs (**A**) or only those that performed both cell and gene therapy (**B**). The location of hubs, spokes, and partner spokes is visualized. Lines connecting hubs to spokes, and spokes to partner spokes reflect the shortest direct distance possible.

First, we mapped the distribution of key facilities that may be involved in a potential hub and spoke model for CGT delivery in Brazil. These included cell processing centers (Supplementary Table 5), health facilities involved in CGT clinical trials (Supplementary Table 6), and potential manufacturers (Supplementary Table 7). The distribution of facilities reflects the health inequality in Brazil, whereby CGT-related facilities were found to be concentrated in major cities in Brazil, especially in São Paulo (Fig. 3). Next, shortlisted hubs (Supplementary Tables 8, 9, and Supplementary Fig. 5), spokes (Supplementary Fig. 6A), and partner spokes (Supplementary Fig. 6B) were assessed against the respective matrices developed previously (Figs. 2, 3). Finally, the locations of the shortlisted hubs, spokes, and partner spokes were populated, and the shortest distance between hub and spoke/spoke and partner spoke was visualized. Two hub and spoke models were simulated in Brazil. The first simulation included all clinical trial sites as hubs (Fig. 4A). This scenario allows for a better population reach; however, designating 35 sites as hubs may be challenging. The second simulation included only clinical trial sites that performed both gene and cell therapy trials as hubs (Fig. 4B). Importantly, in both models, the hubs, spokes, and partner spokes are concentrated in the southeast region of Brazil, where health access and development are most advanced.

**Fig. 5:**
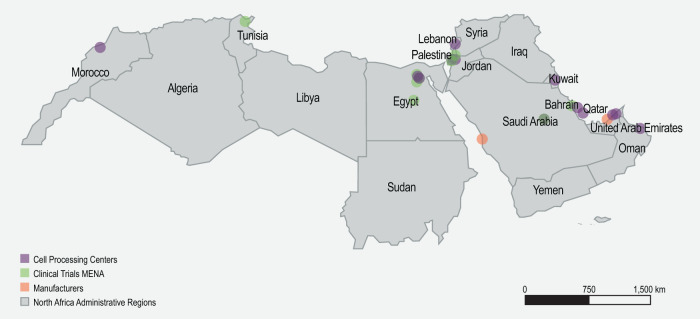
Geographical distribution of facilities involved in CGT clinical trials, cell processing centers, and potential manufacturers in the MENA region. CGT Cell and gene therapy, CPC Cell processing center, MENA Middle East and North Africa.

**Fig. 6:**
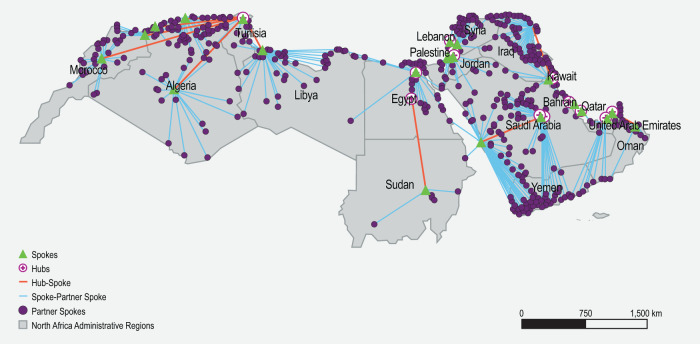
Hub and spoke simulation in the MENA region. The location of hubs, spokes, and partner spokes is visualized. Lines connecting hubs to spokes, and spokes to partner spokes reflect the shortest direct distance possible. MENA, Middle East and North Africa.

Similar to the simulation in Brazil, an extensive mapping exercise was undertaken to identify the potential health facilities that may serve as hubs, spokes, and partner spokes in the MENA region. The identity, location, and geographic distribution of cell processing centers (Supplementary Table 10), potential manufacturers, and health facilities involved in CGT clinical trials (Supplementary Table 11) were populated. Potential CGT facilities were concentrated in the Gulf Cooperation Council countries, like the UAE and Saudi Arabia, where heavy investments in health infrastructure were made during the past decade (Fig. 5). Next, health facilities that were shortlisted as hubs (Supplementary Tables 8 and 12, and Supplementary Fig. 7), spokes (Supplementary Fig. 8A), and partner spokes (Supplementary Fig. 8B) were assessed against the developed matrices. Finally, the location of the shortlisted hubs, spokes, and partner spokes was used to simulate the hub and spoke model in the MENA region (Fig. 6).

## Discussion

Access to CGTs varies massively, with LMICs less likely to benefit, especially given the prohibitive cost associated with such therapies. The preliminary, adaptable roadmap for more equitable CGT delivery in LMICs we propose aims to increase access to CGTs in these countries, but the hub and spoke model is also associated with significant infrastructural, clinical, administrative, and policy requirements. Such requirements are likely to be lacking in most LMICs, and thus the model should be adapted to fit each contextual factor of each country or region. The simulation of the models in Brazil and the MENA region also revealed many opportunities and challenges for future consideration.

The model requires an existing infrastructure that is conducive for expanded CGT services. Brazil has a well-established infrastructure for enabling CGT services and was identified as one of three LMICs to have activity across five advanced therapies/related categories: dedicated government funding, goods and services, pharmaceutical and non-pharmaceutical firms, publications, and academic groups [28]. Further, new centers for CGT manufacturing are under development in Brazil, bolstering local manufacturing of such therapies, and more than 20 CGT clinical trials are underway, setting the stage for increased adoption of CGTs [49]. The proposed model is expected to enhance patient catchment, improve market coverage, and expand patient access to CGTs. Indeed, many of the health centers that were shortlisted as hubs in Brazil are academic medical centers with previous experience in CGT, since they are involved in CGT clinical trials and located near manufacturing facilities in major cities in Brazil. Shortlisted spokes, which have the capacity to collect, process, cryopreserve, and reinfuse, would serve as the treatment home for patients. Shortlisted partner spokes in Brazil, which have apheresis capabilities, are well embedded within communities throughout the country.

The simulation in Brazil has also uncovered challenges that should be considered in future development or potential implementation of the model. First, much of North and West Brazil do not include any hub in the proposed model, which vastly increases distances between hubs and spokes, creating logistical and infrastructural challenges. Second, the model was based on data sets/information from government, nongovernment, pharmaceutical, and literature sources and websites. Many government data sets have not been updated since 2016, and most information needed for full designation of hubs/spokes/partner spokes could not be identified. This may have hindered the identification of potential hubs in North and West Brazil. Engaging the different stakeholders may better inform the potential designation of hubs. Third, the simulation adopts the shortest distance between hub/spoke and spoke/partner spoke. An optimal approach would incorporate the geographic proximity of hubs/spokes/partner spokes, potential clients, distance decay, availability of services relative to demand, road network travel time, and patient catchment into the model. However, building such a model requires extensive data collection across Brazil, and these data sets are currently not available [77–80].

The developed simulation highlights at its core the inequitable capacity and resources for healthcare in the MENA region. The simulated model is polarized: while potential hubs were mostly identified in high-income countries in the Gulf region, especially in Saudi Arabia, not a single spoke was identified in Yemen. Regional health coordination mechanisms are absent in this region, except for the Gulf Cooperation Council countries through the Gulf Health Council, which is hosted in Saudi Arabia and serves six countries. These coordination mechanisms will be essential in planning, executing, and maintaining a hub and spoke model for CGT delivery in the MENA region. Capacity in the MENA region to provide CGTs is severely hindered by a multitude of challenges, especially in countries impacted by political conflict. For example, the total number of clinical trials in Yemen is four, and no bone marrow transplantation facilities could be identified. In Palestine, no clinical trials are registered in international databases [81]. Still, opportunities for implementing the hub and spoke model for CGT delivery in the MENA region are numerous. First, expanding CGT delivery in the MENA region is already underway. In Saudi Arabia, the Ministry of Investment signed a Memorandum of Understanding with Novartis to expand local activities for CGT, including transfer of technology, research and development, and building local capacities [82]. This will ensure better clinical translation and local manufacturing of such therapies. Second, calls for implementing a hub and spoke model for healthcare delivery have been raised, specifically in the Gulf Cooperation Council area [83]. A Saudi BioPharma company named Saudi Vax has signed a letter of intent to establish a facility that will become a CGT hub in the region [60].

The role of regulatory bodies in the administration of CGTs is pivotal. From the approval of clinical trials to the authorization for market access, regulatory authorities play a critical part in the CGT landscape. Within the hub and spoke model, regulatory approval plays a crucial role in defining the responsibilities of different centers, the administration of CGTs, and patient management. The hubs, which are the main healthcare facilities, would need stringent regulatory compliance and accreditation because of the complex nature of the services they offer. Spokes and partner spokes, however, would manage less complex procedures but still need to adhere to the standards set by regulatory bodies. During clinical trials, the regulatory focus is on safety and efficacy, which would require close monitoring of patients and rigorous data collection at all centers. Post-approval, the focus shifts toward broader patient accessibility, long-term safety, and real-world effectiveness. This might require changes in the roles of hubs, spokes, and partner spokes in the model, and also in the processes for patient management and therapy administration.

In addition to establishing robust regulatory frameworks, the implementation of the hub and spoke model necessitates developed infrastructure, with the hubs requiring the most resources and delivering the most complex services. Indeed, many low-income and lower-middle-income countries currently lack the necessary infrastructure, including transplantation centers and apheresis facilities, for the widespread implementation of CGTs. This infrastructural gap presents a substantial challenge for these countries and, without targeted efforts to build capacity and develop necessary infrastructure, could exacerbate existing health inequities. In addition, capacity building is also essential, as healthcare personnel need specialized training to administer CGTs. Partnerships with pharmaceutical companies, academic institutions, and international organizations can help with both capacity building and financing. Finally, innovative financing strategies are needed to manage the high costs associated with CGTs, ensuring that these life-changing therapies are accessible to all who need them.

Although the hub and spoke model can enhance access to CGTs in LMICs, it is essential to acknowledge the disadvantages. One disadvantage is the potential to further exacerbate existing health inequities. As noted in the simulations in Brazil and the MENA region, medical centers that are equipped to handle CGTs are often located in urban or more affluent areas or countries. This can create a disparity in access to these advanced treatments, further advantaging the already privileged population. It is crucial to address this issue and ensure that access to CGTs is equitable and reaches underserved populations. Moreover, investing in CGT in LMICs may have substantial population health implications. While these therapies hold promise for treating specific diseases, diverting financial resources toward their development and implementation could potentially hinder other essential healthcare initiatives. Many LMICs face challenges in providing basic healthcare services, such as newborn screening, cancer screening, and other preventive measures that have a broader population impact. Prioritizing CGTs without addressing these fundamental healthcare needs could perpetuate inequities and neglect the broader health needs of the population.

This study provides insight into the long road ahead to ensure expanded access to CGTs in LMICs. The inclusion of pharmaceutical, clinical, and policy stakeholders from LMICs in the planning, development, and expansion of CGT services is a must. Finally, further investigation into the practical adoption of such a model, focusing on the development of documentation required for health institutions to evaluate readiness for and eventual adoption of the proposed hub and spoke model, is needed.

### Supplementary information

Supplementary Text and Figs

Supplementary Table 1

Supplementary Table 2

Supplementary Table 3

Supplementary Table 4

Supplementary Table 5

Supplementary Table 6

Supplementary Table 7

Supplementary Table 8

Supplementary Table 9

Supplementary Table 10

Supplementary Table 11

Supplementary Table 12

## Data Availability

Data generated for this study are included in the supplementary material. Additional data are available from the corresponding author on reasonable request.

## References

[1] American Society of Gene & Cell Therapy (ASGCT). American Society of Gene & Cell Therapy home page. 2023. https://asgct.org. Accessed 3 April 2023.

[2] U.S. National Library of Medicine. ClinicalTrials.gov home page. 2023. https://clinicaltrials.gov. Accessed 3 April 2023.

[3] Kemler S, Lohr A. Cell & gene therapy investment outlook in 2022 & beyond. 2022. https://www.cellandgene.com/doc/cell-gene-therapies-investment-outlook-in-beyond-0001. Accessed 3 April 2023.

[4] Quinn C, Young C, Thomas J, Trusheim M (2019). Estimating the clinical pipeline of cell and gene therapies and their potential economic impact on the US healthcare system. Value Health.

[5] Elverum K, Whitman M (2020). Delivering cellular and gene therapies to patients: solutions for realizing the potential of the next generation of medicine. Gene Ther.

[6] Cornetta K, Bonamino M, Mahlangu J, Mingozzi F, Rangarajan S, Rao J (2022). Gene therapy access: global challenges, opportunities, and views from Brazil, South Africa, and India. Mol Ther.

[7] Mehler M Operationalizing cell & gene therapy: challenges and solutions. 2022. https://www.cellandgene.com/doc/operationalizing-cell-gene-therapy-challenges-and-solutions-0001. Accessed 3 April 2023.

[8] Doxzen K, Cornetta K, Hongeng S, Kityo C, Mahlangu J, Makani J, et al. Accelerating global access to gene therapies: case studies from low- and middle-income countries. 2022. https://www3.weforum.org/docs/WEF_Accelerating_Global_Access_to_Gene_Therapies_2022.pdf. Accessed 3 April 2023.

[9] Wiseman V, Mitton C, Doyle‐Waters MM, Drake T, Conteh L, Newall AT (2016). Using economic evidence to set healthcare priorities in low‐income and lower‐middle‐income countries: a systematic review of methodological frameworks. Health Econ.

[10] Rattananon P, Anurathapan U, Bhukhai K, Hongeng S (2021). The future of gene therapy for transfusion-dependent beta-thalassemia: the power of the lentiviral vector for genetically modified hematopoietic stem cells. Front Pharmacol.

[11] Porter ME, Lee TH. The strategy that will fix healthcare. 2013. https://hbr.org/2013/10/the-strategy-that-will-fix-health-care. Accessed 3 April 2023.

[12] Elrod JK, Fortenberry JL (2017). The hub-and-spoke organization design revisited: a lifeline for rural hospitals. BMC Health Serv Res.

[13] Dunn L. What the US can learn from healthcare delivery overseas: Q&A with Harvard Business School’s Regina E. Herzlinger. Becker Hospital Review, Online Edition. 2013. https://www.beckershospitalreview.com/hospital-management-administration/what-the-us-can-learn-from-healthcare-delivery-overseas-qaa-with-harvard-business-schools-regina-e-herzlinger.html. Accessed 3 April 2023.

[14] Subramaniam S, Chen J, Wilkerson T, Stevenson L, Kincaid C, Firestone C (2022). Refining the implementation of a hub-and-spoke model for telepain through qualitative inquiry. J Technol Behav Sci.

[15] Demaerschalk B, Miley M, Kiernan T, Bobrow B, Corday D, Wellik K (2009). Stroke telemedicine. Mayo Clin Proc.

[16] Ramachandran V, Alajlani M, Arvanitis TN (2022). A distributed cancer care model with a technology-driven hub-and-spoke and further spoke hierarchy: Findings from a pilot implementation programme in Kerala, India. Asian Pac J Cancer Prev.

[17] Ma Y, Zhang L, Huang X (2014). Genome modification by CRISPR/Cas9. FEBS J..

[18] Harrison RP, Ruck S, Rafiq QA, Medcalf N (2018). Decentralised manufacturing of cell and gene therapy products: learning from other healthcare sectors. Biotechnol Adv.

[19] Srivastava S. Part 2: cell and gene therapy is transforming healthcare. 2020. https://www.cellandgene.com/doc/part-cell-and-gene-therapy-is-transforming-healthcare-0001. Accessed 3 April 2023.

[20] Miesbach W, Chowdary P, Coppens M, Hart DP, Jimenez‐Yuste V, Klamroth R (2021). Delivery of AAV‐based gene therapy through haemophilia centres—a need for re‐evaluation of infrastructure and comprehensive care: a joint publication of EAHAD and EHC. Haemophilia..

[21] Bok A, Noone D, Skouw-Rasmussen N (2022). Key challenges for hub and spoke models of care–a report from the 1st workshop of the EHC think tank on hub and spoke treatment models. J Haemophilia Pract.

[22] Miesbach W, Baghaei F, Boban A, Chowdary P, Coppens M, Hart DP (2022). Gene therapy of hemophilia: hub centres should be haemophilia centres: a joint publication of EAHAD and EHC. Haemophilia..

[23] US Food and Drug Administration. FDA Approves First Gene Therapy for Adults with Severe Hemophilia A. https://www.fda.gov/news-events/press-announcements/fda-approves-first-gene-therapy-adults-severe-hemophilia. Accessed 30 June 2023.

[24] Reiss UM, Mahlangu J, Ohmori T, Ozelo M, Srivastava A, Zhang L (2022). Haemophilia gene therapy: Update on new country initiatives. Haemophilia..

[25] Reiss UM, Zhang L, Ohmori T (2021). Hemophilia gene therapy: New country initiatives. Haemophilia..

[26] Miliotou AN, Papadopoulou LC (2018). CAR T-cell therapy: A new era in cancer immunotherapy. Curr Pharm Biotechnol.

[27] The World Bank. World Bank Open Data. https://data.worldbank.org/country/XO. Accessed 7 August 2023.

[28] Cornetta K, Bonamino M, Mahlangu J, Mingozzi F, Rangarajan S, Rao J (2022). Gene therapy access: global challenges, opportunities and views from Brazil, South Africa, and India. Mol Ther.

[29] RENETA Rede Nacional de Especialistas em Terapias Avançadas home page. 2022. https://www.reneta.org.br. Accessed 3 April 2023.

[30] ا. ل. ص. ح. ة. فريقبوابةوزارة Ministry of Health Saudi Arabia home page. 2023. https://www.moh.gov.sa/Pages/Default.aspx. Accessed 3 April 2023.

[31] TreatSMA. Spinraza access by country. 2019. https://www.treatsma.uk/treatments/spinraza/spinraza-access-by-country/. Accessed 12 April 2023.

[32] Emirates News Agency. Luxturna for treatment of inherited blindness now available: MOHAP. 2019. http://wam.ae/en/details/1395302769783. Accessed 3 April 2023.

[33] Al-awsat A. Saudi Arabia Approves New Drug to Treat Hereditary Loss of Sight. In: Middle-east Arab News Opinion. Saudi Research and Marketing UK Ltd. 2022. https://english.aawsat.com//home/article/1936306/saudi-arabia-approves-new-drug-treat-hereditary-loss-sight. Accessed 3 April 2023.

[34] Genpharm. Genpharm initiates gene therapy for SMA patients in Kuwait & Qatar. 2019. https://www.genpharmservices.com/news/genpharm-initiates-gene-therapy-in-kuwait-qatar-for-its-sma-patients/. Accessed 3 April 2023.

[35] Zaid MA. Egyptian child with SMA receives most expensive medicine in world. 2021. https://www.arabnews.com/node/1820101/food-health. Accessed 3 April 2023.

[36] Hamad A, English AA. Egyptian family raises $2 mln to buy world’s most expensive drug for ill daughter. 2022. https://english.alarabiya.net/variety/2022/06/25/Egyptian-family-raises-2-mln-to-buy-world-s-most-expensive-drug-for-ill-daughter. Accessed 3 April 2023.

[37] Middle East Health. A revolutionary gene therapy first for the Middle East. 2020. https://middleeasthealth.com/focus/interviews/a-revolutionary-gene-therapy-first-for-the-middle-east/. Accessed 3 April 2023.

[38] Al Jalila Children’s Specialty Hospital. Al Jalila Children’s Introduces the First of its Kind Gene Therapy in the UAE. 2020. https://aljalilachildrens.ae/media/al-jalila-children-s-introduces-the-first-of-its-kind-gene-therapy-in-the-uae. Accessed 3 April 2023.

[39] Egypt Today. 2nd Egyptian child with muscular dystrophy injected by Zolgensma. 2021. https://www.egypttoday.com/Article/1/105969/2nd-Egyptian-child-with-muscular-dystrophy-injected-by-Zolgensma. Accessed 3 April 2023.

[40] Ajina R (2022). Abstract P067. Current status of regulatory-approved immunotherapies in Saudi Arabia. Abstract from the American Association for Cancer Research (AACR) Virtual Special Conference: Tumor Immunology and Immunotherapy. Cancer Immunol Res.

[41] BioPhorum. Cell and gene therapy validation challenges. 2021. https://www.biophorum.com/download/cell-and-gene-therapy-validation-challenges. Accessed 3 April 2023.

[42] BioPhorum. CGT considerations when assigning responsibilities during a product transfer between a sponsor and a contract development manufacturing organization. 2021. https://www.biophorum.com/download/cgt-considerations-when-assigning-responsibilities-during-a-product-transfer-between-a-sponsor-and-a-contract-development-manufacturing-organization. Accessed 3 April 2023.

[43] BioPhorum. Commercialization: Gene therapy process map. 2020. https://www.biophorum.com/download/gene-therapy-process-map/. Accessed 3 April 2023.

[44] BioPhorum. Commercialization: Cell therapy process map. 2020. Available from: https://www.biophorum.com/download/cell-therapy-process-map/. Accessed 3 April 2023.

[45] BioPhorum. CGT actors and process maps: Who does what in the supply of different cell and gene therapies. 2022. https://www.biophorum.com/download/cgt-actors-and-process-maps/. Accessed 3 April 2023.

[46] McVey JH, Rallapalli PM, Kemball-Cook G, Hampshire DJ, Giansily-Blaizot M, Gomez K (2020). The European Association for Haemophilia and Allied Disorders (EAHAD) Coagulation Factor Variant Databases: important resources for haemostasis clinicians and researchers. Haemophilia..

[47] Agência Nacional de Vigilância Sanitária. Dados de produção Centros de Processamento Celular. In: Power BI Report. 2020. https://app.powerbi.com/view?r=eyJrIjoiMDE2ZDcwMTUtMmMwNC00NmRhLThlNzQtYzczZGM2NzQwYWRkIiwidCI6ImI2N2FmMjNmLWMzZjMtNGQzNS04MGM3LWI3MDg1ZjVlZGQ4MSJ9. Accessed 3 April 2023.

[48] Rede Nacional de Terapia Celular (RNTC). O que é a RNTC? 2023. http://www.rntc.org.br/a-rntc.html. Accessed 3 April 2023.

[49] McMahon DS, Singer PA, Daar AS, Thorsteinsdóttir H (2010). Regenerative medicine in Brazil: small but innovative. Reg Med.

[50] Sindusfarma. Publications - Highlights. 2023. https://sindusfarma.org.br/publicacoes/destaques. Accessed 3 April 2023.

[51] NUCEL Group of Cellular and Molecular Therapy (Grupo Nucel). Home page. 2021. http://w3nucel.webhostusp.sti.usp.br. Accessed 3 April 2023.

[52] Association for the Advancement of Blood & Biotherapies (AABB). AABB/ABHH Accredited Blood Centers and Transfusion Services. 2023. https://www.aabb.org/standards-accreditation/accreditation/accredited-facilities/aabb-abhh-accredited-blood-centers-and-transfusion-services. Accessed 3 April 2023.

[53] Foundation for the Accreditation of Cellular Therapy (FACT). Search for a FACT Accredited Organization. 2023. https://accredited.factglobal.org. Accessed 3 April 2023.

[54] CnesWeb. Indicadores - Habilitações. 2022. http://cnes2.datasus.gov.br/Mod_Ind_Habilitacoes.asp?VTipo=H. Accessed 3 April 2023.

[55] Parent’s Guide to Cord Blood Foundation. Family Cord Blood Banking in United Arab Emirates. 2023. https://parentsguidecordblood.org/en/family-banking/united-arab-emirates. Accessed 3 April 2023.

[56] Association for the Advancement of Blood & Biotherapies (AABB). Cellular Therapy Facilities. 2023. https://www.aabb.org/standards-accreditation/accreditation/accredited-facilities/cellular-therapy-facilities. Accessed 3 April 2023.

[57] Smart Cells, FamiCord Group. Our Offices. 2021. https://www.smartcells.com/contact/our-offices/. Accessed 3 April 2023.

[58] Government of Dubai. Dubai Cord Blood and Research Center. 2023. https://www.dha.gov.ae/en/facilities/speciality-centers/3. Accessed 3 April 2023.

[59] Matsumoto MM, Dajani R, Matthews KRW (2015). Cord blood banking in the Arab world: current status and future developments. Biol Blood Marrow Transplant.

[60] MilliporeSigma, Merck KGaA. SaudiVax: Regionalizing – and Democratizing – Cell and Gene Therapy. In: The Medicine Maker. 2022. https://themedicinemaker.com/manufacture/saudivax-regionalizing-and-democratizing-cell-gene-therapy-1. Accessed 3 April 2023.

[61] Arab News. Novartis, Sudair Pharma to produce cancer drugs in KSA. 2019. https://www.arabnews.com/node/1490481/jserrors/aggregate. Accessed 3 April 2023.

[62] Abu Dhabi Stem Cell Center (ADSCC). Modern Cellular Therapy. 2022. https://www.adscc.ae/service/car-t-cells. Accessed 3 April 2023.

[63] Sidra Medicine. Advanced Cell Therapy Core. 2023. https://www.sidra.org/research/advanced-cell-therapy-core. Accessed 3 April 2023.

[64] Hamad Medical Corporation. HMC second healthcare organization outside North America to implement innovative gene therapy to treat congenital spinal muscular atrophy. 2019. https://www.hamad.qa/EN/news/2019/November/Pages/HMC-Second-Healthcare-Organization-Outside-North-America-to-Implement-Innovative-Gene-Therapy-to-Treat-Congenital-Spinal-Mu.aspx. Accessed 3 April 2023.

[65] Bioscience Institute, The Group. Bioscience Institute, Genomics and Clinic. 2020. https://bioinst.com/en/the-group-2-2. Accessed 3 April 2023.

[66] EBMT. Centre Membership. 2018. https://www.ebmt.org/ebmt-centre-membership.

[67] Eastern Mediterranean Blood and Marrow Transplantation (EMBMT). Participating Members Directory. 2023. http://www.embmt.org/embmt2/index.php/membership/participating-centers-directory. Accessed 3 April 2023.

[68] History – Al Makassed General Hospital. 2022. https://hospital.makassed.org/en/history. Accessed 3 April 2023.

[69] Center for International Blood & Marrow Transplant Research (CIBMTR). Participating Transplant Centers. 2023. https://cibmtr.org/CIBMTR/About/Our-Impact/Our-Centers. Accessed 3 April 2023.

[70] Shaheen M, Almohareb F, Aljohani N, Ayas M, Chaudhri N, Abosoudah I (2021). Hematopoietic stem cell transplantation in Saudi Arabia between 1984 and 2016: experience from four leading tertiary care hematopoietic stem cell transplantation centers. Hematol Oncol Stem Cell Ther.

[71] Mount Lebanon Hospital University Medical Center. Radiation Oncology – Overview. 2023. http://www.mlh.com.lb/radiation-oncology. Accessed 3 April 2023.

[72] Baldomero H, Aljurf M, Zaidi SZA, Hashmi SK, Ghavamzadeh A, Elhaddad A (2019). Narrowing the gap for hematopoietic stem cell transplantation in the East-Mediterranean/African region: comparison with global HSCT indications and trends. Bone Marrow Transplant.

[73] Bekadja MA, Brahimi M, Osmani S, Yafour N, Krim A, Serradj F (2017). Hematopoietic stem cell transplantation in Algeria. Hematol Oncol Stem Cell Ther.

[74] Attia A, Siala I, Azribi F. General oncology care in Libya. In: Al-Shamsi, Abu-Gheida IH, Iqbal F, Al-Awadi A (eds). Cancer in the Arab World. Springer, Singapore, 2022. pp 133-48.

[75] Ben Othman T, Torjemane L, Abdelkefi A, Lakhal A, Ladeb S, Ben Hamed L (2008). Allogeneic hematopoietic stem cell transplantation in Tunisia. Bone Marrow Transplant.

[76] QGIS home page. 2023. https://qgis.org/en/site. Accessed 3 April 2023.

[77] Crooks VA, Schuurman N (2012). Interpreting the results of a modified gravity model: examining access to primary health care physicians in five Canadian provinces and territories. BMC Health Serv Res.

[78] Tanser F (2006). Methodology for optimising location of new primary health care facilities in rural communities: a case study in KwaZulu-Natal, South Africa. J Epidemiol Community Health.

[79] Joseph AE, Bantock PR (1982). Measuring potential physical accessibility to general practitioners in rural areas: a method and case study. Soc Sci Med.

[80] Watson DE, Krueger H, Mooney D, Black C. Planning for renewal: mapping primary health care in British Columbia. Centre for Health Services and Policy Research (CHSPR) University of British Columbia, Vancouver, 2005.

[81] U.S. Library of Medicine. Studies By Topic – Location. ClinicalTrials.gov. 2023. https://clinicaltrials.gov/ct2/search/browse?brwse=locn_cat_ME. Accessed 3 April 2023.

[82] International Business Magazine. MISA signs up MoU with the Novartis aimed at pharma expansion in KSA. 2022. https://intlbm.com/2022/06/03/misa-signs-up-mou-with-the-novartis-aimed-at-pharma-expansion-in-ksa/. Accessed 3 April 2023.

[83] Arab Health. Driving change. 2017. https://www.arabhealthonline.com/magazine/en/latest-issue/3/driving-change.html. Accessed 3 April 2023.

