# Supplementary material for: A practical approach for adoption of a hub and spoke model for cell and gene therapies in low- and middle-income countries: framework and case studies
Source: Gene Ther. 2023 Oct 30;31(1-2):1–11. doi: 10.1038/s41434-023-00425-x (PMC10788266; doi:10.1038/s41434-023-00425-x)
Supplement: Supplementary file 1 — Supplementary Text and Figs [file 41434_2023_425_MOESM1_ESM.pdf]

SUPPLEMENTARY MATERIAL

Supplemental text

List of MENA countries [1]

MENA (the Middle East and North Africa) Region: covers 24 countries, namely, the 21 members of the Arab League (Algeria, Bahrain, Djibouti, Egypt, Iraq, Jordan, Kuwait, Lebanon, Libya, Mauritania, Morocco, Oman, Palestine, Qatar, Saudi Arabia, Somalia, Sudan, Syria, Tunisia, the United Arab Emirates, and Yemen), as well as Iran, Israel, and Turkey.

Framework of CGT Stakeholders Expanded Description

Core direct stakeholders include beneficiaries, therapeutic centers, treatment coordination actors, and manufacturing stakeholder actors, in addition to payers and regulatory agencies as indirect stakeholders. Beneficiaries may be patients or donors. In allogenic therapy, a healthy donor (sometimes the patient) provides cells used to manufacture the therapeutic product [2]. Patients may also provide cells for diagnostic purposes that will inform production of a therapeutic product or for direct use in cell manufacturing. In addition, patients receive treatment with this therapeutic product, usually in the form of an infusion [3, 4].

Depending on the country and CGT type, a therapeutic center may be a specialist disease center, a center of excellence, a local or regional hospital, an academic medical center, or an independent or private practice. First, a therapeutic center collects a specimen from patients or donors for diagnostic or cell manufacturing purposes. Second, the therapeutic center performs pretesting on patients to inform eligibility. For example, antibody testing is done to determine if an individual has preexisting antibodies that may prevent therapy from being successful [5]. Third, the therapeutic center initiates the entire supply chain through ordering the therapy from the manufacturer and ensuring the ability to pay [6]. Fourth, the therapeutic center prepares the patient for therapy, which may include chemotherapies and immunosuppressants. Finally, the therapeutic center administers the treatment to the patient, mostly via infusion.

Treatment coordination of CGTs generally requires planning, patient operations, accounting, an orchestration platform, and courier services. Planning ensures that the raw material and manufacturing capacity required are available, based on anticipated demand of products [3]. Patient operations implies coordination with therapeutic centers, ensuring up-to-date information regarding the various points of supply throughout the treatment process, and handling issues. Accounting coordinates with payers, manufacturers, and therapeutic centers throughout and after the treatment process to ensure that agreements, invoices, and payments are in place. The orchestration platform ensures proper scheduling, quality, and risk assessment of the supply process. Finally, the courier services deliver products and material through the supply chain,

ensuring that storage, movement, and delivery remain under the proper conditions and adhere to the appropriate timelines [3, 7].

The main roles of manufacturing are to analyze specimens, prepare cells, and develop and test therapeutic products. Sequencing labs, using next-generation sequencing, are an essential part of this manufacturing and may be involved in cell line/viral vector testing and cell/viral bank production, as well as quality control for therapy delivery [8]. Cell preprocessing facilities, usually located close to therapeutic centers, are important for preparing patient specimens to be moved through the CGT pipeline. Vector manufacturing facilities may be focused on plasmid or genetic vectors, as both genetic and cell-based therapies require plasmids to deliver the gene product. Plasmid manufacturing is essential for creation and testing of the sequences of nucleic acids (DNA or RNA) that will be used in manufacturing therapeutic products. Genetic vectors, which may be personalized or produced in bulk and include viral or mRNA vectors, will carry the new genetic sequence that will be used in the next manufacturing step [9]. Importantly, the coordination between one or more payers, including health insurance companies, private individuals or organizations, and national health agencies, initiates the CGT therapeutic process 10]. Finally, regulatory agencies are in place to authorize and monitor the CGT pipeline, which may include initial authorization to produce a therapeutic product or the development of processes to monitor manufacturing, supply, and therapy administration.

To showcase the interaction between the core CGT stakeholders, we populated process flow mapping for the various types of gene and cell therapies, adapted from BioPhorum's CGT actors processes [3], including made for stock genetic vector (**Fig. 1**), personalized gene therapy (**Supplementary Fig. 2**), allogenic cell therapy (**Supplementary Fig. 3**), and autologous cell therapy (**Supplementary Fig. 4**).

Hub and Spoke Model Expanded Description

A CGT hub is a state-of-the-art academic medical center with proven expertise, research abilities, and practice with CGTs (**Fig. 2A**). Although patients may be closely managed at the spoke, decisions regarding CGTs will be a collaborative process between hub and spoke personnel to ensure optimal patient outcomes. The therapeutic center at the CGT hub has the capacity to screen, diagnose, and treat patients undergoing CGT, and should have the treatment capacity for all categories of CGT. To do this, the hub should harbor an in-house apheresis and cryopreservation capacity, with a greater number of intensive care unit beds in case of adverse events related to CGT. Specifically, hubs should have the facilities to prioritize CGT patients while possessing the needed capacity (e.g., cryopreservation and apheresis) to deliver the required therapy [11]. These facilities should have an existing and established logistics and supply chain, as well as storage capacity (e.g., refrigeration, deep cold storage, reconstitution). The manufacturers should share space with or be located near the therapeutic center for a hub. These therapeutic centers will include a sequencing lab, preprocessing, plasmid manufacturing, genetic vector manufacturing, and/or cell manufacturing facilities. Importantly, the CGT hub incorporates an academic and therapeutic center that, in coordination with biotech and pharmaceutical companies, should establish a manufacturing network that can provide cell/tissue

processing services or automated CGT manufacturing services [12]. The collaboration between the above actors also results in a shorter path from research to clinical application of CGTs.

The CGT therapeutic center at the hub must be accredited by agencies based in the country where the hub is located. Manufacturing should be compliant with global regulations, like the Good Manufacturing Practice. The CGT hub should also be able to offer its spokes connected services for compliance assistance, and have an existing CGT registry or work to develop one. These registries will be key in recording real-world data on therapies used, treatment conditions and evaluation, longitudinal patient data, and other information [13]. The hub will also participate in clinical trials and longitudinal long-term observational studies. Registries can also be a part of the regulatory process. For example, inclusion in registries is required for CGTs that follow the Regenerative Medicine and Advanced Therapy regulatory pathway under the US Food and Drug Administration [14]. Relevant training for physicians to provide up-to-date CGT care should be in place, especially since different CGTs require different standards of care, and CGT clinical developments are fast paced. Notably, health care professionals (HCPs) cannot work in silos when working within a hub and spoke system. For example, cell therapy patients will likely require complex care before, during, and after therapy and may be seen by multiple HCPs at the spoke and the hub. These patients may also require immune system ablation, putting them at greater risk for infection [15]. As such, coordination between health care workers at both the hub and spoke is key. In a hub, the orchestration platform expands its role to provide end-to-end supply visibility. To do this, the orchestration platform will comprise two functional units. First, a visibility and monitoring unit includes a dashboard that provides real-time contextual visibility of all events and activities and monitors product and material flow across the hub and spoke. This unit will ensure that the chain of identity and chain of custody are maintained. This is particularly relevant for autologous therapy, to ensure that the patient receives their product and not someone else's. Second, an information technology harmonization unit works with information technology departments in the hubs and spokes to facilitate and standardize collection, breakdown, and analysis of patient, tissue, transport, and manufacturing outcomes. This unit also strives to implement artificial intelligence and smart automation into the supply and value chain systems.

The CGT spoke aims to increase patient access and decrease overall therapy costs. A CGT spoke is a medical facility, which might be a local hospital, a health center, or a municipal hospital. Hub apheresis facilities will not have enough machines, beds, or personnel to meet the expected increase in CGT delivery [16] (**Fig. 2B**). As such, spokes ought to house apheresis centers. The spoke should be able to administer at least one type of CGT and is responsible for ensuring that the therapeutic practices and requirements are in place at its facility. The spoke should at least have a cell/tissue processing facility to collect, preserve, and ship cells to the hub and/or manufacturing facilities because for certain therapies, it is key to optimize the identification, sourcing, collection, and delivery of donor starting material. Starting manufacturing material may include whole blood, stimulated apheresis, bone marrow, and core blood units, among others. Spoke collection centers should have the ability to properly collect, store, and prepare

shipments through well-developed apheresis and shipping services. Importantly, the spokes provide referral routes to hubs and are considered the home center for patients, where patients will undergo continuous treatment and rigorous follow-up. Treatment protocols and process flows should be standardized throughout all spokes in coordination with the hub. The spoke is also a donation center, where donors provide cells or other specimen, which are then shipped to manufacturing facilities, potentially at the CGT hub. Medical history for donors and patients should be properly collected by the health care team, which also includes a focal HCP. At the hub, HCPs should be trained to diagnose and prepare patients and administer treatment. HCPs may also oversee patient information sharing with the hub and partner spokes when relevant. Like hubs, a treatment coordinator should be stationed at spokes to organize patient schedules and coordinate with partner spokes for proper specimen collection and shipping.

The CGT partner spoke is a supporting facility and may include outpatient centers, clinics, apheresis centers, blood collection centers, or actors (e.g., Red Cross or Red Crescent) (**Fig. 2C**). The current and projected volumes of CGT and their corresponding trials are much greater than existing accredited centers can currently handle [17]. Nonaccredited centers are therefore needed to screen patients and perform apheresis. This is where partner spokes complement the function of spokes, as they serve as screening, referral, and collection centers. They operate in small communities without full accreditation. These partner spokes optimize cell transit time, increase access to clinical trial participants, and simplify the logistics process. Distributed blood collection centers will provide access to additional resources to extract and process cells and tissues. Outpatient centers and clinics can be partners to increase participation in clinical trials by widening access to potential participants, especially in remote areas in LMICs. They can also aid in increasing referrals to spokes for CGT. Consequently, partner spokes, especially outpatient centers and clinics, should have qualified personnel to diagnose and refer patients for CGT. Importantly, donors and patients can provide raw material through collection centers, including Red Cross and Red Crescent, that are spread widely throughout LMICs. These centers can collect relevant data on patients and donors and coordinate with spokes for proper shipping and initiation of the manufacturing process. This also requires proper scheduling for specimen collection and sharing patient information with spokes and hubs.

SUPPLEMENTARY FIGURES

Supplementary Fig. 1. Process flow of made-for-stock genetic vectors. Green font and arrows represent communication among stakeholders. Red font and arrows represent physical handling between stakeholders.

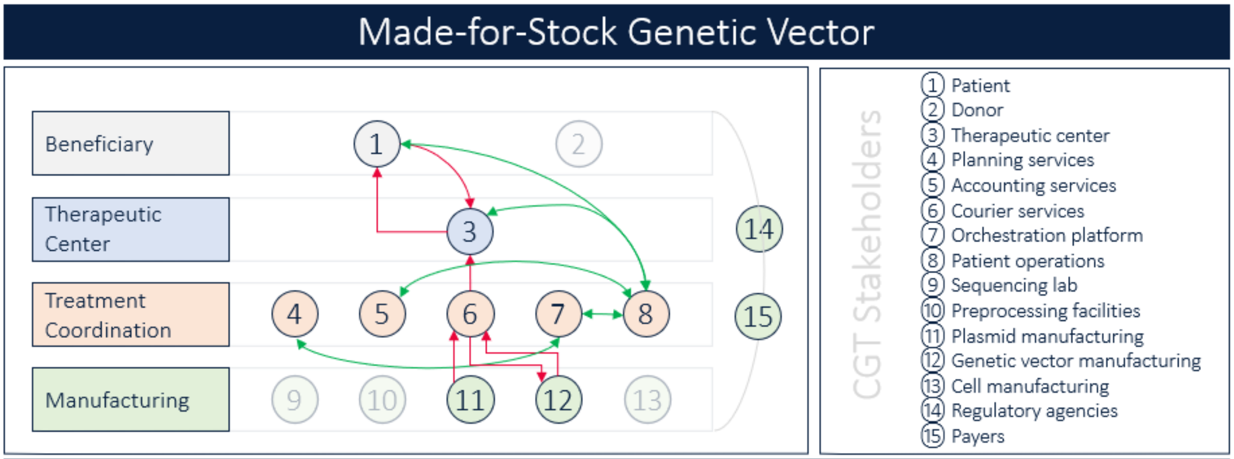

- Therapeutic center ③ pretests patient ① for suitability of therapy and places order.
- Patient operations ⑧ communicates with therapeutic center ③ for orders, then communicates with accounting ⑤ and the orchestration platform ⑦ to update the therapeutic center ③ and patient ① on the supply process.
- Planning services ④ communicates with the orchestration platform ⑦ to plan the supply process.
- The orchestration platform ⑦ coordinates with all actors in the supply process, managing supply risk, quality, and scheduling.
- Accounting services ⑤ tracks costs and payments throughout the supply and treatment processes.
- Plasmid manufacturing ⑪ produces and tests the plasmids, which are then shipped to genetic vector manufacturing ⑫ to produce and test the genetic vector.
- The courier services ships the genetic vector to the therapeutic center ③, where it will be infused into the patient ①.
- Regulatory agencies ⑭ may be involved in authorizing the manufacturing of products and monitoring for quality control and compliance.
- Finally, payers ⑮ (multiple or single) process payments (one or more).

Supplementary Fig. 2. Process flow of personalized gene therapies. Green font and arrows represent communication among stakeholders. Red font and arrows represent physical handling between stakeholders.

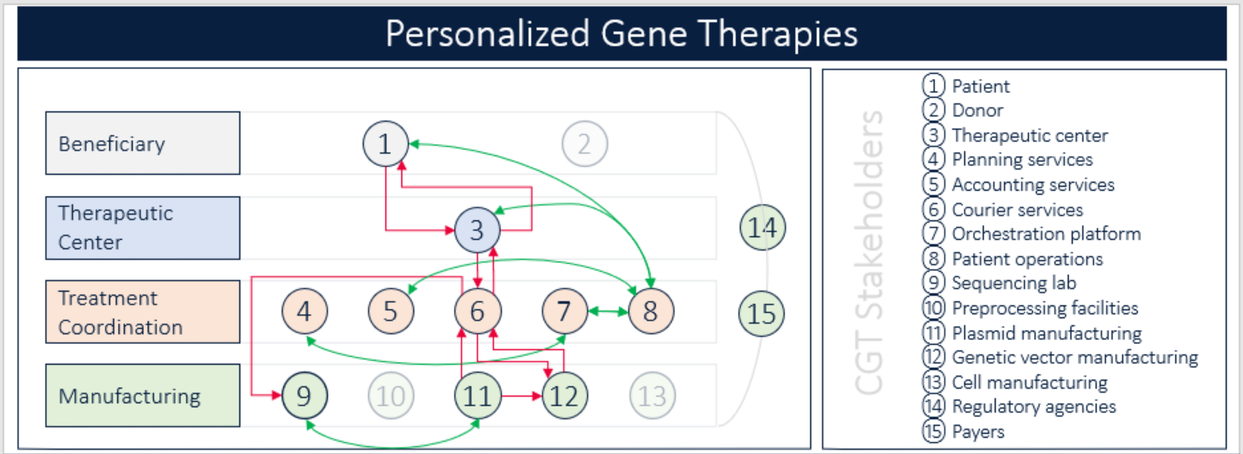

- Patient (1) provides specimen to therapeutic center (3), which then ships the specimen through courier services (6) to the sequencing lab (9) to analyze the specimen.
- Patient operations (8) communicates with therapeutic center (3) for orders, then communicates with accounting (5) and the orchestration platform (7) to update the therapeutic center (3) and patient (1) on the supply process.
- Based on the analysis, the sequencing lab (9) coordinates with plasmid manufacturing (11) to create plasmids that are shipped through courier services (6) to genetic vector manufacturing (12) to produce a genetic vector.
- Courier services ships the genetic vector to the therapeutic center (3), where it will be infused into the patient (1).
- Planning services (4) communicates with the orchestration platform (7) to plan the supply process.
- Orchestration platform (7) coordinates with the sequencing lab (9), planning services (4), genetic vector manufacturing (12), and patient operations (8) thoughout the supply process, managing supply risk, quality, and scheduling.
- Accounting services (5) tracks costs and payments throughout the supply and treatment processes.
- Regulatory agencies (14) may be involved in authorizing the manufacturing of products and monitoring for quality control and compliance.
- Payers (15) (multiple or single) process payments (one or more).

Supplementary Fig. 3. Process flow of allogenic cell therapies. Green font and arrows represent communication among stakeholders. Red font and arrows represent physical handling between stakeholders.

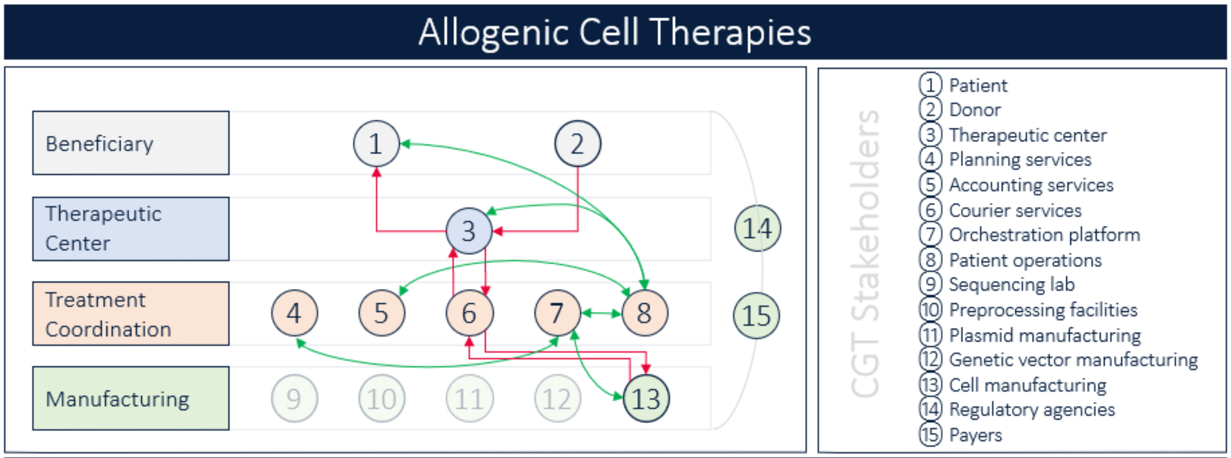

- Donor (2) donates specimen to therapeutic center (could be donor center) (3), which then ships the specimen through courier services (6) to cell manufacturing (9) to manufacture cell product, which may include generating and testing cell bank.
- Planning services (4) communicates with orchestration platform (7) to plan the supply process.
- Orchestration platform (7) coordinates with planning services (4), cell manufacturing (13), and patient operations (8) thoughout the supply process, managing supply risk, quality, and scheduling.
- The courier services ships the therapeutic product to the therapeutic center (3), where it will be infused into the patient (1).
- Accounting services (5) tracks costs and payments throughout the supply and treatment processes.
- Regulatory agencies (14) may be involved in authorizing the manufacturing of products and monitoring for quality control and compliance.
- Payers (15) (multiple or single) process payments (one or more).

Supplementary Fig. 4. Process flow of autologous cell therapies. Green font and arrows represent communication among stakeholders. Red font and arrows represent physical handling between stakeholders

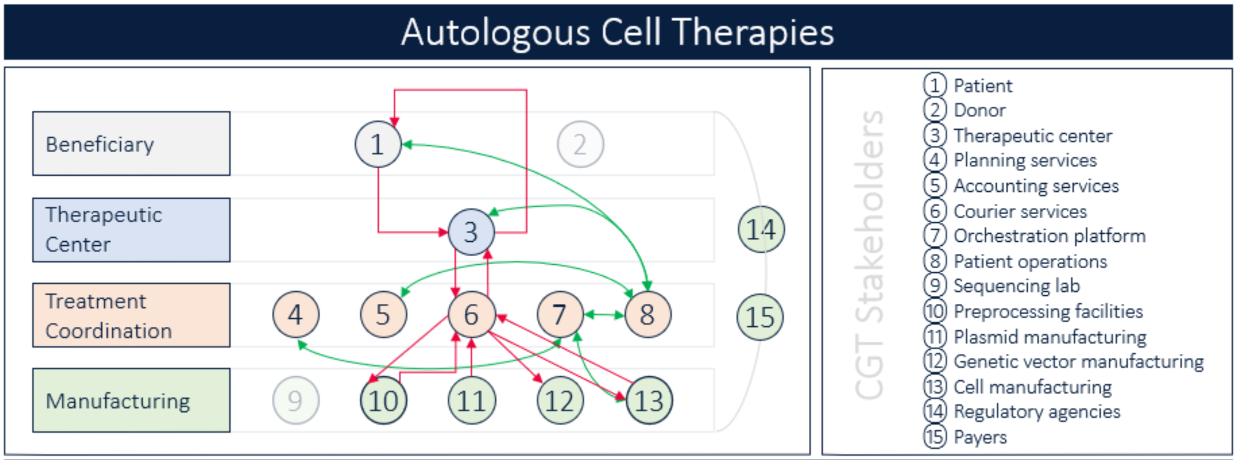

- Specimen from patient ① is collected at therapeutic center ③, which then ships the specimen through courier services ⑥ to cell preprocessing facility ⑩ to cells for shipment to manufacturing facility ⑬.
- Plasmids ⑪ containing genetic sequence are made and then shipped ⑥ to genetic vector manufacturing ⑫ to produce vector, which is then used to manipulate patient cells at cell manufacturing facility ⑬.
- Modified cells are shipped ⑥ to therapeutic center ③ and infused back into the patient ①.
- Planning services ④ communicates with orchestration platform ⑦ to plan the supply process.
- Orchestration platform ⑦ coordinates with cell preprocessing facility ⑩, genetic vector manufacturing ⑫, cell manufacturing ⑬, and patient operations ⑧ thoughout the supply process, managing supply risk, quality, and scheduling.
- Accounting services ⑤ tracks costs and payments throughout the supply and treatment processes.
- Regulatory agencies ⑭ may be involved in authorizing the manufacturing of products and monitoring for quality control and compliance.
- Payers ⑮ (multiple or single) process payments (one or more).

Supplementary Fig. 5. Shortlisted CGT hubs in Brazil were assessed against core hub criteria developed. Each criterion was either determined to be available (green), absent (red), or couldn't be determined. Shortlisted hub identification and criteria codes are used in the matrix.

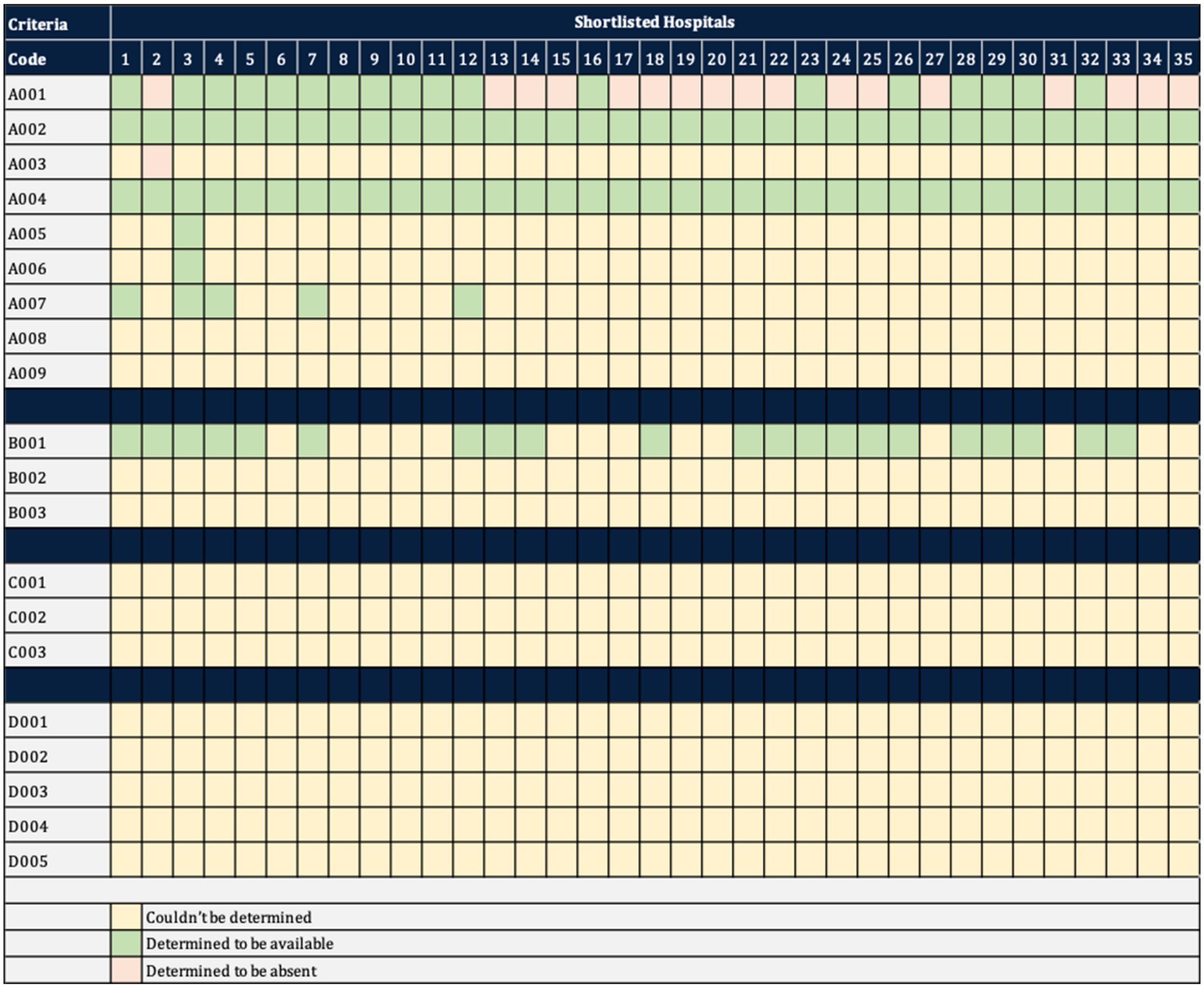

Supplementary Fig. 6. The core capacities of shortlisted CGT spoke (A) and partner spoke (B) in Brazil were either determined to be available (green) or could not be determined (yellow). All spokes and partner spokes were considered as one unit, respectively.

A- Core Capacities of a CGT Spoke	
**A. Clinical and Research Capacities**	**Code**
A medical center with minimal or no experience in CGT	A001
Have the clinical capacity to administer at least one type of CGT therapy	A002
Capacity to screen for and perform clinical trials	A003
Houses an apheresis center and a cell/gene processing facility	A004
Performs regular follow up (3 to 6 months) on patients undergoing CGT	A005
Ability to screen, diagnose, order, store, prepare, and administer CGT	A006
Capacity to perform longitudinal data collection and evaluation, in collaboration with hubs and partner spokes	A007
**B. Manufacturing Services**	
Cohabited or physically close to a Sequencing lab	B001
The sequencing services require sequencing and quality focal points	B002
**C. Human Resources**	
Qualified health care professionals who can order, store, prepare, and administer at least one type of CGT	C001
Ability to train partner spokes on proper collection and shipping	C002
Employs a treatment coordinator, with limited roles compared to hub	C003
Employs a Patient operations professional, accountant, and logistics coordinator, as well as payer/insurance coordinator	C004
**D. Other Services**	
Houses, or is associated with, a well-developed shipping service	D001
Secure IT infrastructure to share patient data with hub and receive patient data from partner spokes	D002

Couldn't be determined

Determined to be available

B- Core Capacities of a CGT Partner Spoke
May include outpatient centers, clinics, or apheresis or blood collection centers
Capacity to perform apheresis when relevant
Houses the facilities to screen participants for clinical trials and refer to spokes
Partner spoke is embedded within communities, especially in remote locations
Secure IT infrastructure to share patient data with spoke
Qualified health care professionals that screen patients, potentially collect blood/apheresis, and refer patients to spokes
Employs a treatment coordinator, patient operations professional, and a logistics coordinator, with limited roles.

Couldn't be determined

Determined to be available

Supplementary Fig. 7. Shortlisted CGT hubs in the MENA region were assessed against core hub criteria developed. Each criterion was either determined to be available (green), absent (red), or couldn't be determined. Shortlisted hub identification and criteria codes are used in the matrix.

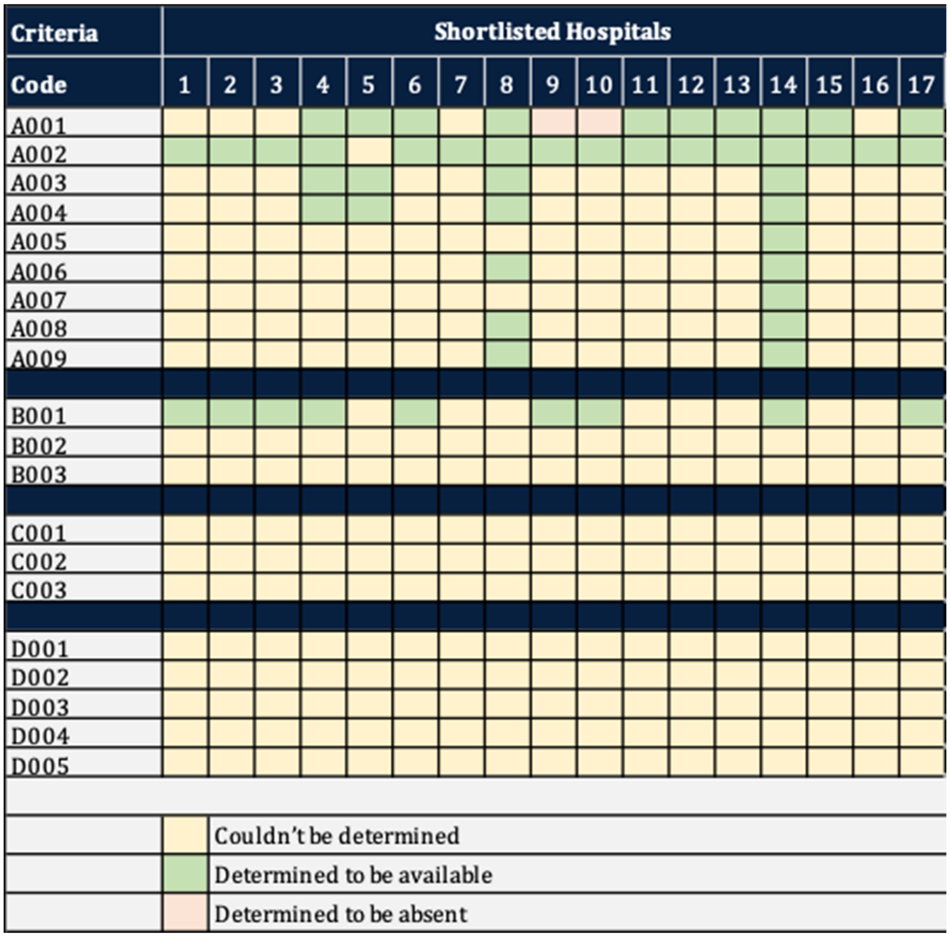

Supplementary Fig. 8. The core capacities of shortlisted CGT spokes (A) and partner spokes (B) in the MENA region were either determined to be available (green) or couldn't be determined (yellow). All spokes and partner spokes were considered as one unit.

A- Core Capacities of a CGT Spoke	
**A. Clinical and Research Capacities**	
A medical center with minimal or no experience in CGT	
Have the clinical capacity to administer at least one type of CGT therapy	
Capacity to screen for and perform clinical trials	
Houses an apheresis center and a cell/gene processing facility	
Performs regular follow up (3 to 6 months) on patients undergoing CGT	
Ability to screen, diagnose, order, store, prepare, and administer CGT	
Capacity to perform longitudinal data collection and evaluation, in collaboration with hubs and partner spokes	
**B. Manufacturing Services**	
Cohabited or physically close to a Sequencing lab	
The sequencing services require sequencing and quality focal points	
**C. Human Resources**	
Qualified health care professionals who can order, store, prepare, and administer at least one type of CGT	
Ability to train partner spokes on proper collection and shipping	
Employs a treatment coordinator, with limited roles compared to hub	
Employs a Patient operations professional, accountant, and logistics coordinator, as well as payer/insurance coordinator	
**D. Other Services**	
Houses, or is associated with, a well-developed shipping service	
Secure IT infrastructure to share patient data with hub and receive patient data from partner spokes	

	Couldn't be determined
	Determined to be available

B- Core Capacities of a CGT Partner Spoke	
May include outpatient centers, clinics, or apheresis or blood collection centers	
Capacity to perform apheresis when relevant	
Houses the facilities to screen participants for clinical trials and refer to spokes	
Partner spoke is embedded within communities, especially in remote locations	
Secure IT infrastructure to share patient data with spoke	
Qualified health care professionals that screen patients, potentially collect blood/apheresis, and refer patients to spokes	
Employs a treatment coordinator, patient operations professional, and a logistics coordinator, with limited roles.	

	Couldn't be determined
	Determined to be available

REFERENCES

1. El-Erian MA, Fischer S. Is MENA a region? The scope for regional integration. Economic and Political Impediments to Middle East Peace. Palgrave Macmillan, London, 2000.
2. Klein HG. Cellular gene therapy: an overview. J Clin Apher. 1994;9:139–141.
3. BioPhorum. CGT actors and process maps: Who does what in the supply of different cell and gene therapies. 2022. https://www.biophorum.com/download/cgt-actors-and-process-maps/. Accessed 3 April 2022.
4. Mayo Clinic. Gene therapy. 2017. https://www.mayoclinic.org/tests-procedures/gene-therapy/about/pac-20384619. Accessed 3 April 2022.
5. Colella P, Ronzitti G, Mingozzi F. Emerging issues in AAV-mediated in vivo gene therapy. Mol Ther Methods Clin Dev. 2018;8:87–104.
6. Vineti. When the patient is the product – some key challenges in cell and gene therapies. 2019. https://vineti.com/resources/blog/when-the-patient-is-the-product-some-key-challenges-in-cell-and-gene-therapies/. Accessed 3 April 2023.
7. Accenture. Accelerating the culture cure. 2022. https://www.accenture.com/us-en/insights/life-sciences/cellandgenetherapy. Accessed 3 April 2023.
8. Genewiz. Cell and gene therapy solutions. Research areas. 2023. https://www.genewiz.com/en/Public/Research-Areas/Cell-and-Gene-Therapy. Accessed 3 April 2023.
9. Bulcha JT, Wang Y, Ma H, Tai PWL, Gao G. Viral vector platforms within the gene therapy landscape. Signal Transduct Target Ther. 2021;6:53.

10. Loche A, Paolucci N, Peters N, Van der Veken L. A call to action: Opportunities and challenges for CGTs in Europe. 2021. https://www.mckinsey.com/industries/life-sciences/our-insights/a-call-to-action-opportunities-and-challenges-for-cgts-in-europe. Accessed 3 April 2023.

11. Elverum K, Whitman M. Delivering cellular and gene therapies to patients: solutions for realizing the potential of the next generation of medicine. Gene Ther. 2020;27:537–544.

12. Harrison RP, Ruck S, Rafiq QA, Medcalf N. Decentralised manufacturing of cell and gene therapy products: learning from other healthcare sectors. Biotechnol Adv. 2018;36:345–357.

13. Alliance for Regenerative Medicine. ARM and NAMCP publish recommendations to increase patient access in joint study, "Roadmap for navigating the provider side of cell and gene therapy (CGT) patient access in US managed care." 2020. https://alliancerm.org/press-release/arm-and-namcp-publish-recommendations-to-increase-patient-access-in-joint-study-roadmap-for-navigating-the-provider-side-of-cell-and-gene-therapy-cgt-patient-access-in-us-managed-care/. Accessed 3 April 2023.

14. U.S. Food and Drug Adminstration. Regenerative Medicine Advanced Therapy Designation. 2023. https://www.fda.gov/vaccines-blood-biologics/cellular-gene-therapy-products/regenerative-medicine-advanced-therapy-designation. Accessed 3 April 2023.

15. McGuirk J, Waller EK, Qayed M, Abhyankar S, Ericson S, Holman P, et al. Building blocks for institutional preparation of CTL019 delivery. Cytotherapy. 2017;19:1015–1024.

16. Bryne J. Alleviating CGT bottlenecks: standards needed to ensure volumes, quality of starting material. 2021. https://www.biopharma-

reporter.com/Article/2021/11/12/Alleviating-CGT-bottlenecks-Standards-needed-to-ensure-volumes-quality-of-starting-material. Accessed 3 April 2023.

17. Mehler M. Operationalizing cell & gene therapy: challenges and solutions. 2022. https://www.cellandgene.com/doc/operationalizing-cell-gene-therapy-challenges-and-solutions-0001. Accessed 3 April 2023.
