# Supplementary material for: A practical approach for adoption of a hub and spoke model for cell and gene therapies in low- and middle-income countries: framework and case studies
Source: Gene Ther. 2023 Oct 30;31(1-2):1–11. doi: 10.1038/s41434-023-00425-x (PMC10788266; doi:10.1038/s41434-023-00425-x)
Supplement: Supplementary file 2 — Supplementary Table 1 [file 41434_2023_425_MOESM2_ESM.pdf]

**Supplementary Table 1. Capacities matrix for beneficiaries in a CGT hub and spoke model**

| Beneficiaries | | | | |
|---|---|---|---|---|
| **Actor** | **Characteristic** | **Hub** | **Spoke** | **Partner Spoke** |
| Donor | Donates cells or other specimen | ✓ | ✓ | ✓ |
| | Provides accurate information on preexisting conditions or changes in medical history | ✓ | ✓ | ✓ |
| Patient | Gives specimen | ✓ | ✓ | ✓ |
| | Accepts preparatory treatments. Receives treatment | ✓ | ✓ | X |
| | Maintains appointments, confirms personal information is accurate | ✓ | ✓ | ✓ |
| | Screened for clinical trials | ✓ | ✓ | ✓ |
| | Enrolled in clinical trials | ✓ | ✓ | X |

CGT, cell and gene therapy.
✓, has capacity; X, does not have capacity.
Note: Cells highlighted in green indicate new roles within a hub and spoke model that are typically not present in existing models of CGT delivery.
