# Supplementary material for: A practical approach for adoption of a hub and spoke model for cell and gene therapies in low- and middle-income countries: framework and case studies
Source: Gene Ther. 2023 Oct 30;31(1-2):1–11. doi: 10.1038/s41434-023-00425-x (PMC10788266; doi:10.1038/s41434-023-00425-x)
Supplement: Supplementary file 3 — Supplementary Table 2 [file 41434_2023_425_MOESM3_ESM.pdf]

Supplementary Table 2. Capacities matrix for therapeutic center in a CGT hub and spoke model

Therapeutic Center				
**Actor**	**Characteristic**	**Hub**	**Spoke**	**Partner Spoke**
Health care professional	Qualified and undertakes relevant training	✓	✓	X
	Collects patient medical history<br>Advises patient on and prescribes therapy selection<br>Prepares patient (or donor) for specimen collection and final treatment	✓	✓	X
	Takes specimen and delivers to shipping partner<br>Administers treatment	✓	✓	X
	Shares patient information with CGT hub	X	✓	✓
	Coordinates with partner spoke regarding patient information	X	✓	X
Treatment coordinator	Places the order, organizes the schedule for patient preparation and treatment	✓	✓	X
	Books the manufacturing slot	✓	X	X
	Organizes the scheduling of each stage of specimen collection	✓	✓	✓
	Coordinates training for partner spokes on proper collection and shipping	X	✓	X
	Coordinates training for spokes on proper treatment, collection, and shipping	✓	X	X
	Develops standardized treatment protocols and process flows for all treatment centers	✓	X	X

CGT, cell and gene therapy.

✓, has capacity; X, does not have capacity.

Note: Cells highlighted in green indicate new roles within a hub and spoke model that are typically not present in existing models of CGT delivery.
