# Supplementary material for: A practical approach for adoption of a hub and spoke model for cell and gene therapies in low- and middle-income countries: framework and case studies
Source: Gene Ther. 2023 Oct 30;31(1-2):1–11. doi: 10.1038/s41434-023-00425-x (PMC10788266; doi:10.1038/s41434-023-00425-x)
Supplement: Supplementary file 4 — Supplementary Table 3 [file 41434_2023_425_MOESM4_ESM.pdf]

Supplementary Table 3. Capacities matrix for treatment coordination in a CGT hub and spoke model

Treatment Coordination				
**Actor**	**Characteristic**	**Hub**	**Spoke**	**Partner Spoke**
Supply chain planner	Ensures supply of raw materials, components, resources, etc., at various time points in the end-to-end process	✓	X	X
Patient operations professional	Liaises with therapeutic centers on behalf of patients; accepts, tracks, and fulfills the therapeutic product order	✓	✓	✓
	Liaises with hub to ensure the fulfilment of CGT	✓	X	X
Patient outcomes follow-up	Liaises with therapeutic centers to track long-term patient outcomes and keeps the payer/insurance coordinator informed	✓	✓	X
	Liaises with CGT registrar and collects longitudinal data on patients	✓	✓	X
	Shares longitudinal data with hub registrar	✓	✓	X
CGT registrar	Records real-world data on therapies used, treatment conditions and evaluations, and longitudinal data on patients	✓	X	X
Visibility and monitoring unit	Monitors product and material flow across the hub and spoke<br>Ensures that the chain of identity and chain of custody are maintained	✓	X	X
IT harmonization unit	Works with IT departments in the hubs and spokes to facilitate and standardize collection, breakdown, and analysis of patient, tissue, transport, and manufacturing outcomes	✓	X	X
E2E delivery accountable	Coordinates all batches that are time critical, E2E, vein-to-vein, across all supply partners and shipping facilities	✓	X	X
E2E quality accountable	Coordinates the quality of all batches, bringing together the input of all supply partners and shipping facilities	✓	X	X
Logistics coordinator	Manages delivery of items from treatment center to manufacturing facility, between facilities, and from manufacturing facility back to the treatment center<br>Deals with any schedule changes	✓	X	X
	Manages delivery of blood/apheresis collected at partners to hub or spoke	X	X	✓
	Manages delivery of items to spoke from manufacturing and from spoke to manufacturing in the hub	X	✓	X

Accountant	Accrues and tracks orders, incoming patient material (if billed separately), works in progress, finished goods (likely using job costing for personalized medicine), and patient treatment receipt (to recognize revenue)	✓	✓	X
Payer/insurance coordinator	Requests and pursues appropriate payment according to the agreed schedule, including any outcome-based factors, liaising with the payer organizations (sometimes over the long term)	✓	✓	X

CGT, cell and gene therapy; E2E, end-to-end; IT, information technology.

✓, has capacity; X, does not have capacity.

Note: Cells highlighted in green indicate new roles within a hub and spoke model that are typically not present in existing models of CGT delivery.
