# Supplementary material for: A practical approach for adoption of a hub and spoke model for cell and gene therapies in low- and middle-income countries: framework and case studies
Source: Gene Ther. 2023 Oct 30;31(1-2):1–11. doi: 10.1038/s41434-023-00425-x (PMC10788266; doi:10.1038/s41434-023-00425-x)
Supplement: Supplementary file 5 — Supplementary Table 4 [file 41434_2023_425_MOESM5_ESM.pdf]

Supplementary Table 4. Capacities matrix for manufacturing in a CGT hub and spoke model

Manufacturing				
**Actor**	**Characteristic**	**Hub**	**Spoke**	**Partner Spoke**
Sequencing focal point	Analyzes the patient specimen using NGS to produce data used in the manufacturing process (e.g., to determine which plasmids to use)	✓	✓	X
Cell preprocessing production coordinator	Offers available slots for cell preprocessing to the control tower	✓	X	X
Cell preprocessing production planner	Plans the preprocessing details for all incoming batches to prepare for shipment to the main manufacturing facility	✓	X	X
Plasmid production coordinator	Fulfills the delivery schedules agreed upon with the supply chain planner and reports any issues or delays	✓	X	X
Plasmid production planner	Plans the manufacturing details for all incoming batches	✓	X	X
Genetic manufacturing production coordinator	Offers available slots for personalized cell manufacturing or fulfills the bulk delivery schedules agreed upon with the supply chain planner and reports any issues or delays	✓	X	X
Genetic manufacturing production planner	Plans the manufacturing details for all batches of genetic vectors	✓	X	X
Cell production coordinator	Offers available slots for personalized cell manufacturing or fulfills the bulk delivery schedules agreed upon with the supply chain planner reports any delays or issues	✓	X	X
Cell production planner	Plans the manufacturing detail for all batches of cells	✓	X	X
Quality responsible	Ensures the output of this part of the process meets the appropriate quality criteria	✓	✓	X

CGT, cell and gene therapy; NGS, next-generation sequencing.

✓, has capacity; X, does not have capacity.
