# Supplementary material for: A practical approach for adoption of a hub and spoke model for cell and gene therapies in low- and middle-income countries: framework and case studies
Source: Gene Ther. 2023 Oct 30;31(1-2):1–11. doi: 10.1038/s41434-023-00425-x (PMC10788266; doi:10.1038/s41434-023-00425-x)
Supplement: Supplementary file 6 — Supplementary Table 5 [file 41434_2023_425_MOESM6_ESM.pdf]

Supplementary Table 5. Name, home institution, and state of cell technology centers and cell processing centers in Brazil

Cell Technology Centers		
**Name**	**Home Institution**	**State**
Instituto Nacional de Cardiologia		RJ
Fundação Hemocentro de Ribeirão Preto	Faculdade de Medicina de Ribeirão Preto da Universidade de São Paulo	SP
Centro de Biotecnologia E Terapia Celular	Hospital São Rafael	BA
Núcleo de Tecnologia Celular	Pontifícia Universidade Católica do Paraná (PUCPR)	PR
PUC do Paraná		
Núcleo de Terapia Celular e Molecular	Faculdade de Medicina da Universidade de São Paulo	SP
**Cell Processing Centers**		
**Name**	**Home Institution**	**State**
Biotec Hemoterapia	Biotec Hemoterapia	SP
CCB - Centro de Criogenia Brasil	Centro de Criogenia Brasil	SP
Centro de Biotecnologia e Terapia Celular do Hospital São Rafael (CBTC-HSR)	Hospital São Rafael	BA
Centro de Hematologia do Vale	Centro de Hematologia do Vale	SP
Centro de Tecidos Biológicos de Minas Gerais-Cetebio / Fundação Hemominas	Fundação Hemominas	MG
Colsan Associação Beneficente de Coleta de Sangue	Colsan	SP
CPC da Fundação Hemocentro de Brasília	Fundação Hemocentro de Brasília	DF
CPC DA Irmandade Santa Casa de Misericórdia de São Paulo	Distrito Federal	SP
CPC da União Oeste Paranaense de Combate Ao Câncer (UOPECCAN)	UOPECCAN	PR
CPC da Unidade de Hemoterapia e Hematologia do Samaritano	Hospital Samaritano	SP
CPC do Banco de Sangue de Caxias do Sul	Condomínio Centro Médico Pasteur	RS
CPC do Banco de Sangue Paulista (HHEMO)	Banco de Sangue Paulista (HHEMO)	SP
CPC do Hemocentro de Botucatu (HCFMB)	Hospital das Clínicas de Botucatu	SP
CPC do Hemocentro de Santa Catarina (HEMOSC)	Hemocentro de Santa Catarina (HEMOSC)	SC
CPC do Hemocentro do Ceará (HEMOCE)	Hemocentro do Ceará	CE
CPC do Hemonúcleo Regional Jaú	Hospital Amaral Carvalho	SP
CPC do Hospital A C Camargo	Hospital A C Camargo	SP
CPC do Hospital Angelina Caron	Hospital Angelina Caron	PR
CPC do Hospital Araújo Jorge - Associação de Combate ao Câncer em Goiás	Hospital Araújo Jorge - Associação de Combate ao Câncer em Goiás	GO
CPC do Hospital de Amor de Barretos (Fundação Pio XII)	Hospital de Amor de Barretos (Fundação Pio XII)	SP
CPC do Hospital de Base de São José do Rio Preto	Hospital de Base de São José do Rio Preto	SP
CPC do Hospital de Clínicas de Porto Alegre	Hospital de Clínicas de Porto Alegre	RS

CGT, cell and gene therapy; CPC, cell processing center.
