# Supplementary material for: A practical approach for adoption of a hub and spoke model for cell and gene therapies in low- and middle-income countries: framework and case studies
Source: Gene Ther. 2023 Oct 30;31(1-2):1–11. doi: 10.1038/s41434-023-00425-x (PMC10788266; doi:10.1038/s41434-023-00425-x)
Supplement: Supplementary file 7 — Supplementary Table 6 [file 41434_2023_425_MOESM7_ESM.pdf]

Supplementary Table 6. CGT clinical trials in Brazil: therapeutic product, CGT type, sponsor, health facilities involved, and cities

Therapeutic product	CGT type	Sponsor	Health facilities	City
Nestacell	Cell therapy	Azidus Brasil	Hospital Vera Cruz	SP
			Hospital de Barueri	SP
			Instituto do Coração do Hospital das Clínicas da FMUSP	SP
			UNIFESP	SP
Cilta-cel	Cell therapy	Janssen	Hospital São Rafael	BA
			A.C. Camargo Cancer Center	SP
			Hospital Israelita Albert Einstein	SP
COVI-MSC	Cell therapy	Sorrento Therapeutics	Hospital São Rafael S.A. - Instituto D'Or de Pesquisa e Ensino	BA
			Santa Casa de Misericórdia da Bahia (Hospital Santa Izabel)	BA
			Saraiva & Berlinger Ltda. - EPP (IPECC)	SP
			CECIP JAU - Centro de Estudos Clínicos do Interior Paulista Ltda.	SP
			CEMEC - Centro Multidisciplinar de Estudos Clínicos Ltda. - EPP	SP
			Impar Serviços Hospitalares S/A (Hospital Nove de Julho)	SP
PBKR03	Gene therapy	Passage Bio, Inc.	Hospital de Clínicas de Porto Alegre	RS
PBFT02	Gene therapy	Passage Bio, Inc.	Hospital das Clínicas da Universidade Federal de Minas Gerais (UFMG)	MG
			Hospital das Clínicas da Faculdade de Medicina da Universidade de São Paulo (HCFMUSP)	SP

BIIB067	RNA therapy	Biogen	Associação Cruz Verde	SP
TNK-tPA	Gene therapy	University of Calgary	Hospital de Clínicas de Botucatu	SP
			Instituto Hospital de Base do Distrito Federal	DF
			Hospital Universitário Maria Aparecida Pedrossian	MS
			Hospital Celso Ramos Florianopolos	SC
			Hospital Geral de Fortaleza	CE
			Clínica Neurologica e Neurocirurgica de Joinville Ltda	SC
			Porto Alegre Hospital	RS
			Santa Casa de Porto Alegre	RS
			Hospital de Clínicas de Ribeirão Preto	SP
			Americas Medical City	RJ
			Hospital das Clínicas da Faculdade de Medicina da Universidade de São Paulo	SP
			Hospital São Paulo UNIFESP	SP
			Irmandade da Santa Casa de Misericordia de Sao Paulo	SP
			Hospital Estadual Central	ES
PBGM01	Gene therapy	Passage Bio	Hospital de Clínicas de Porto Alegre	RS
NK Cells	Cell therapy	Hospital de Clínicas de Porto Alegre	Hospital de Clínicade Porto Alegre	RS

CGT, cell and gene therapy.
