# Supplementary material for: A practical approach for adoption of a hub and spoke model for cell and gene therapies in low- and middle-income countries: framework and case studies
Source: Gene Ther. 2023 Oct 30;31(1-2):1–11. doi: 10.1038/s41434-023-00425-x (PMC10788266; doi:10.1038/s41434-023-00425-x)
Supplement: Supplementary file 8 — Supplementary Table 7 [file 41434_2023_425_MOESM8_ESM.pdf]

Supplementary Table 7. Name, associated institution, and city of CGT manufacturers in Brazil

CGT Manufacturers in Brazil		
**Name**	**Associated Institution**	**City**
Excellion Serviços Biomédicos		RJ
Farmacore		SP
Cryopraxis		RJ
Hygeia Biotech	Universidade de Rio de Janeiro	RJ
Cellpraxis		SP
Nucell Assistência Técnica	Universidade de São Paulo	SP
Associação Paranaense de Cultura – PUC-PR Campus Curitiba		SP
Biomarin Brasil Farmacêutica Ltda		SP
Cellavita Pesquisas Científicas Ltda		SP
Medspace do Brasil Pesquisa Clinica Ltda		SP
Novartis Biosciências S.A.		SP
Pharm Olam Serviços Clinicos Ltda		SP
Sindus Farma		SP
Janssen Cilag Farmacêuticos		SP
Azidus Brasil		SP
Pfizer Brazil		SP
Biogen Brasil Produtos Farmacêuticos		SP
Hospital de Clínicas de Porto Alegre		SP

CGT, cell and gene therapy.
