# Supplementary material for: A practical approach for adoption of a hub and spoke model for cell and gene therapies in low- and middle-income countries: framework and case studies
Source: Gene Ther. 2023 Oct 30;31(1-2):1–11. doi: 10.1038/s41434-023-00425-x (PMC10788266; doi:10.1038/s41434-023-00425-x)
Supplement: Supplementary file 9 — Supplementary Table 8 [file 41434_2023_425_MOESM9_ESM.pdf]

Supplementary Table 8. Coding of core capacities of a CGT hub, with each criterion assigned a distinct code

Core Capacities of a CGT Hub	
**A. Clinical and Research Capacities**	**Code**
An academic medical center located in major cities	A001
Previous experience in delivering CGT/leading clinical trials	A002
Has a high number of ICU beds	A003
In-house apheresis and cryopreservation capacity	A004
Existing and established logistics and supply chain, and storage capacity (e.g., refrigeration, deep cold storage, reconstitution)	A005
Ability to screen, diagnose, order, store, prepare, and administer CGT	A006
Accredited by international organizations for delivering CGT	A007
Capacity to perform longitudinal data collection and evaluation, in collaboration with spokes	A008
Immunosuppression protocols in place	A009
**B. Manufacturing Services**	**Code**
Sharing space with or physically close to a sequencing lab	B001
Ability to establish a manufacturing network that can provide cell/tissue processing services or automated CGT manufacturing services	B002
The manufacturing services require cell preprocessing production coordinator, cell preprocessing production planner, plasmid production coordinator, plasmid production planner, genetic manufacturing production coordinator, genetic manufacturing production planner, cell production coordinator, cell production planner, and quality responsible	B003
**C. Human Resources**	**Code**
Qualified health care professionals who can order, store, prepare, and administer at least one type of CGT	C001
Ability to train spokes on proper treatment, collection, and shipping	C002
Employs a treatment coordinator, supply chain planner, patient operations professional, CGT registrar, visibility and monitoring unit, information technology harmonization unit, E2E delivery accountable, E2E quality accountable, logistics coordinator, accountant, payer/insurance coordinator	C003
**D. Other Services**	**Code**
Houses an orchestration platform, with developed IT infrastructure for proper monitoring and evaluation, as well as data sharing and analysis	D001
Existing, or ability to establish, a CGT registry	D002
Houses an information technology harmonization	D003
Houses, or is associated with, a well-developed shipping service	D004
Regulatory services department to ensure proper regulatory oversight and compliance assistance for spokes	D005

CGT, cell and gene therapy; E2E, end-to-end; ICU, intensive care unit; IT, information technology.
