# Supplementary material for: A practical approach for adoption of a hub and spoke model for cell and gene therapies in low- and middle-income countries: framework and case studies
Source: Gene Ther. 2023 Oct 30;31(1-2):1–11. doi: 10.1038/s41434-023-00425-x (PMC10788266; doi:10.1038/s41434-023-00425-x)
Supplement: Supplementary file 10 — Supplementary Table 9 [file 41434_2023_425_MOESM10_ESM.pdf]

Supplementary Table 9. Coding of shortlisted CGT hubs in Brazil. Each hospital was assigned an ID and the type of clinical trial was noted.

CGT HUB Shortlisted Hospitals		
**ID**	**Hospital name**	**Clinical trial type**
1	Hospital de Clínicas de Porto Alegre	Cell and gene therapy
2	Parana's Hematology and Hemotherapy Center (HEMEPAR)	Gene therapy
3	Hospital Israelita Albert Einstein	Cell and gene therapy
4	Hospital das Clínicas da Faculdade de Medicina da Universidade de São Paulo UNIFESP	Cell and gene therapy
5	Irmandade da Santa Casa de Misericórdia de São Paulo	Gene therapy
6	Hospital das Clínicas Botucatu - Unesp	Gene therapy
7	Hospital das Clínicas da Faculdade de Medicina de Ribeirão Preto da Universidade de São Paulo	Cell and gene therapy
8	Hospital de Base de São José do Rio Preto	Cell therapy
9	Fundação Pio XII, Hospital de Câncer de Barretos	Cell therapy
10	Instituto Hospital de Base do Distrito Federal	Gene therapy
11	Hospital São Rafael Pronto Socorro	Cell and gene therapy
12	Campinas Estadual University (UNICAMP) / Campinas Hemocentro	Gene therapy
13	Associação Cruz Verde	Gene therapy
14	Centro de Hematologia e Hemoterapia do Espírito Santo	Gene therapy
15	Clinica Neurologica e Neurocirurgica de Joinville Ltda	Gene therapy
16	Complexo Hospitar Universitário Prof. Edgard Santos (HUPES)	Gene therapy
17	Hospital Celso Ramos Florianopolos	Gene therapy
18	Hospital de Barueri	Cell therapy
19	Hospital Estadual Central	Gene therapy
20	Hospital Geral de Fortaleza	Gene therapy
21	Hospital Mario Covas	Cell therapy

22	Hospital Sírio Libanês	Gene therapy
23	Hospital Universitário Maria Aparecida Pedrossian	Gene therapy
24	Hospital Vera Cruz	Cell therapy
25	Instituto Estadual de Hematologia Arthur de Siqueira Cavalcanti	Gene therapy
26	Instituto Nacional de Câncer José Alencar Gomes da Silva - INCA	Cell therapy
27	Santa Casa de Porto Alegre	Gene therapy
28	American Medical City	Gene therapy
29	Hospital São Paulo UNIFESP	Cell and gene therapy
30	Hospital das Clínicas da Universidade Federal de Minas Gerais (UFMG)	Gene therapy
31	CEMEC - Centro Multidisciplinar de Estudos Clínicos Ltda. - EPP	Cell therapy
32	Impar Serviços Hospitalares S/A (Hospital Nove de Julho)	Cell therapy
33	A.C. Camargo Cancer Center	Cell therapy
34	Santa Casa de Misericórdia da Bahia (Hospital Santa Izabel)	Cell therapy
35	CECIP JAU	Gene therapy

CGT, cell and gene therapy.
