# Supplementary material for: A practical approach for adoption of a hub and spoke model for cell and gene therapies in low- and middle-income countries: framework and case studies
Source: Gene Ther. 2023 Oct 30;31(1-2):1–11. doi: 10.1038/s41434-023-00425-x (PMC10788266; doi:10.1038/s41434-023-00425-x)
Supplement: Supplementary file 11 — Supplementary Table 10 [file 41434_2023_425_MOESM11_ESM.pdf]

Supplementary Table 10. Facility names and countries of cell processing centers in the MENA region

Potential Cell Processing Centers in the MENA Region			
**Facility**	**Country**	**Facility**	**Country**
Future Health	Bahrain	Biovault	Lebanon
LifeCell Arabia	Bahrain	Biomax	Lebanon
Biovault	Bahrain	Lifeline	Lebanon
Cells4Life	Bahrain	Reviva	Lebanon
Cryoviva Bahrain	Bahrain	Future Health	Morocco
Smart Cells	Egypt	Smart Cells	Oman
Cell Safe Cord Blood Bank	Egypt	CellSave	Oman
Cells4Life	Egypt	cryoviva	Oman
CellSave	Egypt	Sultan Qaboos University Hospital	Oman
Cell Safe	Egypt	BabyCord	Palestine
StemPlus	Egypt	Virgin Health Bank	Qatar
Center for Stem Cell Research & Regenerative Medicine	Egypt	Future Health Biobank	Qatar
Smart Cells	Jordan	Cells4Life	Qatar
BabyCord	Jordan	CellSave	Qatar
Future Health	Jordan	Smart Cells	Saudi Arabia
KHCC	Jordan	Dubai Cord Blood and Research Center (DCRC)	UAE
Future Health	Kuwait	Medcells	UAE
LifeCell Arabia	Kuwait	Smart Cells	UAE
Smart Cells	Kuwait	Future Health	UAE
Cells4Life	Kuwait	Family Bank LifeCell	UAE
CellSave	Kuwait	Cells4Life Middle East	UAE
Smart Cells	Lebanon	Cryoviva Gulf	UAE
health	Lebanon		

CGT, cell and gene therapy; MENA, Middle East and North Africa; UAE, United Arab Emirates.
