# Supplementary material for: A practical approach for adoption of a hub and spoke model for cell and gene therapies in low- and middle-income countries: framework and case studies
Source: Gene Ther. 2023 Oct 30;31(1-2):1–11. doi: 10.1038/s41434-023-00425-x (PMC10788266; doi:10.1038/s41434-023-00425-x)
Supplement: Supplementary file 12 — Supplementary Table 11 [file 41434_2023_425_MOESM12_ESM.pdf]

Supplementary Table 11. CGT clinical trials in the MENA region: therapeutic product, CGT type, sponsor, health facilities, and countries

CGT Clinical Trials in the MENA Region				
**Therapeutic product**	**CGT type**	**Sponsor**	**Health facilities**	**Country**
Nusinersen	Gene therapy	Biogen	Saint George University Hospital Medical Center	Lebanon
			King Fahad Specialist Hospital –Dammam	Saudi Arabia
			King Faisal Specialist Hospital & Research Center	Saudi Arabia
FLT201	Gene therapy	Freeline Therapeutics	La Rabta Hospital	Tunisia
hMaxi-K	Gene therapy	Urovant Sciences Gmbh	Dasman Diabetes Institute	Kuwait
PF-07055480	Gene therapy	Pfizer	King Fahad Specialist Hospital – Dammam	Saudi Arabia
rAAV2-VMD2-hMERTK	Gene therapy	King Khaled Eye Specialist Hospital	King Fahad Specialist Hospital –Dammam	Saudi Arabia
PF-06838435	Gene therapy	Pfizer	King Fahad Specialist Hospital – Dammam	Saudi Arabia
hLB-001	Gene therapy	Logicbio Therapeutics	King Abdullah Specialist Children Hospital	Saudi Arabia
Prove	Cell and gene therapy	Ain Shams University	Ain Shams University	Egypt
MSCs therapy	Cell therapy	Aljazeera Hospital	Aljazeera (Al Gazeera) Hospital	Egypt
PRP and cord blood	Cell therapy	Aljazeera Hospital	Aljazeera Hospital	Egypt
Lipogems	Cell therapy	American University of Beirut Medical Center	American University of Beirut	Lebanon
JNJ-68284528	Cell therapy	Janssen	King Faisal Specialist Hospital & Research Center	Saudi Arabia
MSCs therapy	Cell therapy	University of Jordan	University of Jordan	Jordan
SLN124	Gene therapy	Silence Therapeutics	Jordan University Hospital	Jordan
			King Hussein Cancer Center	Jordan
			Irbid Specialty Hospital	Jordan
MSCs	Cell therapy	University of Jordan	University of Jordan	Jordan
MSC-PLGA	Cell therapy	University of Jordan	University of Jordan	Jordan
WJ-MSCs	Cell therapy	University of Jordan	University of Jordan	Jordan

CGT, cell and gene therapy; MENA, Middle East and North Africa; MSC, mesenchymal stem cells; PLGA, poly(lactic-co-glycolic acid); WJ-MSC, Wharton's jelly-Mesenchymal stem cell.
