# Supplementary material for: A practical approach for adoption of a hub and spoke model for cell and gene therapies in low- and middle-income countries: framework and case studies
Source: Gene Ther. 2023 Oct 30;31(1-2):1–11. doi: 10.1038/s41434-023-00425-x (PMC10788266; doi:10.1038/s41434-023-00425-x)
Supplement: Supplementary file 13 — Supplementary Table 12 [file 41434_2023_425_MOESM13_ESM.pdf]

Supplementary Table 12. Coding of shortlisted CGT hubs in the MENA region. Each hospital was assigned an ID and the type of clinical trial was noted.

CGT HUB Shortlisted Hospitals		
**ID**	**Hospital Name**	**Country**
1	Cleveland Clinic Abu Dhabi	UAE
2	ADSCC	UAE
3	Bioscience Clinic Middle East	UAE
4	Hamad General Hospital	Qatar
5	Sidra Medicine	Qatar
6	Ain Shams University	Egypt
7	Aljazeera Hospital	Egypt
8	American University of Beirut	Lebanon
9	Dasman Diabetes Institute	Kuwait
10	Irbid Speciality Hospital	Jordan
11	Jordan University Hospital	Jordan
12	King Abdullah Specialist Children Hospital	Saudi Arabia
13	King Fahad Specialist Hospital – Dammam	Saudi Arabia
14	King Faisal Specialist Hospital & Research Center, Riyadh	Saudi Arabia
15	King Hussein Cancer Center	Jordan
16	La Rabta Hospital	Tunisia
17	Saint George University Hospital Medical Center	Lebanon

CGT, cell and gene therapy; MENA, Middle East and North Africa; UAE, United Arab Emirates.
